# Integrated volatile metabolomic and transcriptomic analysis provides insights into the regulation of floral scents between two contrasting varieties of *Lonicera japonica*

**DOI:** 10.3389/fpls.2022.989036

**Published:** 2022-09-12

**Authors:** Jianjun Li, Xinjie Yu, Qianru Shan, Zhaobin Shi, Junhua Li, Xiting Zhao, Cuifang Chang, Juanjuan Yu

**Affiliations:** ^1^Green Medicine Biotechnology Henan Engineering Laboratory, Engineering Technology Research Center of Nursing and Utilization of Genuine Chinese Crude Drugs in Henan Province, College of Life Sciences, Henan Normal University, Xinxiang, China; ^2^Henan International Joint Laboratory of Agricultural Microbial Ecology and Technology, College of Life Sciences, Henan Normal University, Xinxiang, China; ^3^State Key Laboratory Cell Differentiation and Regulation, College of Life Sciences, Henan Normal University, Xinxiang, Henan, China

**Keywords:** *Lonicera japonica* Thunb., floral scent, metabolomics, transcriptomics, volatile

## Abstract

*Lonicera japonica* Thunb., belonging to the Caprifoliaceae family, is an important traditional Chinese medicinal plant. The *L. japonica* flower (LJF) is widely used in medicine, cosmetics, drinks, and food due to its medicinal and sweet-smelling properties. Considerable efforts have been devoted to investigating the pharmacological activities of LJF; however, the regulatory mechanism of the floral scents remains unknown. We previously selected and bred an elite variety of *L. japonica* var. *chinensis* Thunb. called ‘Yujin2’, which has a strong aroma and is used in functional drinks and cosmetics. In order to reveal the regulatory mechanism of the floral scents of LJF, volatile metabolomic and transcriptomic analyses of the LJF at the silver flowering stage of ‘Yujin2’ (strong aroma) and ‘Fengjin1’ (bland odor) were performed. Our results revealed that a total of 153 metabolites and 9,523 genes were differentially regulated in LJF between ‘Yujin2’ and ‘Fengjin1’. The integrated analysis of omics data indicated that the biosynthetic pathways of terpenoids (i.e., monoterpenoids, including geraniol and alpha-terpineol; sesquiterpenoids, including farnesol, farnesal, and alpha-farnesene; triterpenoid squalene), tryptophan and its derivatives (methyl anthranilate), and fatty acid derivatives, were major contributors to the stronger aroma of ‘Yujin2’ compared to ‘Fengjin1’. Moreover, several genes involved in the terpenoid biosynthetic pathway were characterized using quantitative real-time PCR. These results provide insights into the metabolic mechanisms and molecular basis of floral scents in LJF, enabling future screening of genes related to the floral scent regulation, such as alpha-terpineol synthase, geranylgeranyl diphosphate synthase, farnesyl pyrophosphate synthase, anthranilate synthase, as well as transcription factors such as MYB, WRKY, and LFY. The knowledge from this study will facilitate the breeding of quality-improved and more fragrant variety of *L. japonica* for ornamental purpose and functional beverages and cosmetics.

## Introduction

In plants, floral scents play vital roles in attracting pollinators, deterring pathogens and parasites, and serving as signals in response to biotic and abiotic stresses (Schiestl, 2015). In addition, floral scents are important commercial traits of ornamental plants and are also economically important for quality in the food, drink, perfume, cosmetic, and pharmaceutical industries (Mostafa et al., 2022). Floral scents consist of a mixture of volatile organic compounds (VOCs), which are lipophilic and are characterized by low molecular weights and high melting points. To date, more than 1,700 floral VOCs have been identified, which are categorized as terpenoids, phenylpropanoids/benzenoids, fatty acid derivatives, and amino acid derivatives. Over the past decade, a large number of studies on floral VOCs have improved our understanding of their functions, biosynthesis, and regulation. Currently, the research on floral scents is mainly focused on common ornamental plants, such as rose, orchid, and tulip (Mostafa et al., 2022). However, the constituents and abundances of the VOCs in floral scents vary widely among plants. The mechanisms underlying the floral scent regulation of certain important medicinal and edible plant species remain to be elucidated (Zhu et al., 2019; Mostafa et al., 2022).

*Lonicera japonica* Thunb., an important traditional Chinese medicinal plant, is a perennial semi-evergreen twining species of the Caprifoliaceae family member that is cultivated worldwide, particularly in Asian countries such as China, Japan, and Korea. The *L. japonica* flower (LJF) has been prescribed in traditional Chinese medicine for the treatment of infections, fever, sores, swelling, and influenza for thousands of years (Shang et al., 2011). Recently, LJF has also been demonstrated to inhibit influenza A viruses and COVID-19 (Zhou et al., 2015, 2020). Moreover, LJF is also widely used in cosmetics, food, beverages, and ornamental groundcovers due to its medicinal properties and sweet-smelling or attractively colored flowers (Wang et al., 2016).

Owing to its vital role, a considerable amount of research has been devoted to analyzing the chemical constituents and pharmacological activities of LJF (Yoo et al., 2008; Wang et al., 2016; Li et al., 2020). Among these, some progress has been made in analyzing the volatile components of LJF from different origins (Du et al., 2015), parts (Yan et al., 2020), treatments (Cai et al., 2013), and flowering stages (Wang et al., 2009). However, comparative analysis of VOC profiles among different *L. japonica* varieties is very scarce so far. The correlation between characteristic aroma pattern and VOC profile of LJF remains elusive.

‘Yujin2’, an elite variety of *L. japonica* var. *chinensis* Thunb. that was selected and bred by our group, has strong cold and drought resistances, high yield, as well as early and long flowering, with large flower buds. The LJF of ‘Yujin2’ has strong aroma and no bitterness and is purple-red at the budding stage and has thus become a good choice for flower tea and herbal drinks (Li et al., 2019). In contrast, the LJF of ‘Fengjin1’, the predominant variety of *L. japonica* Thunb. grown in Henan, has a bland odor, bitter taste, is green-to-white at the budding stage, and is mainly used in medicines. Therefore, ‘Yujin2’ and ‘Fengjin1’, two contrasting varieties with distinct aroma concentration, are excellent materials for researching the correlation between floral scent and VOC profile of LJF.

Omics technologies, especially transcriptomics, are effective technologies for studying the mechanisms underlying plant growth and development. Considerable numbers of transcriptomic studies have been conducted on LJF. However, these previous studies mainly focused on the mechanism of bioactive constituent biosynthesis during flower development (Li et al., 2019; Yang et al., 2019; Wang et al., 2020; Xia et al., 2021) and in response to salt stress (Cai et al., 2021, 2022) and light stress (Fang et al., 2020). Knowledge of the mechanism underlying the accumulation of VOCs associated with floral scents remains limited. The decoded genome of *L. japonica* has provided valuable information for research into gene function and transcriptomic analysis (Pu et al., 2020; Xiao et al., 2021).

**Figure 1:**
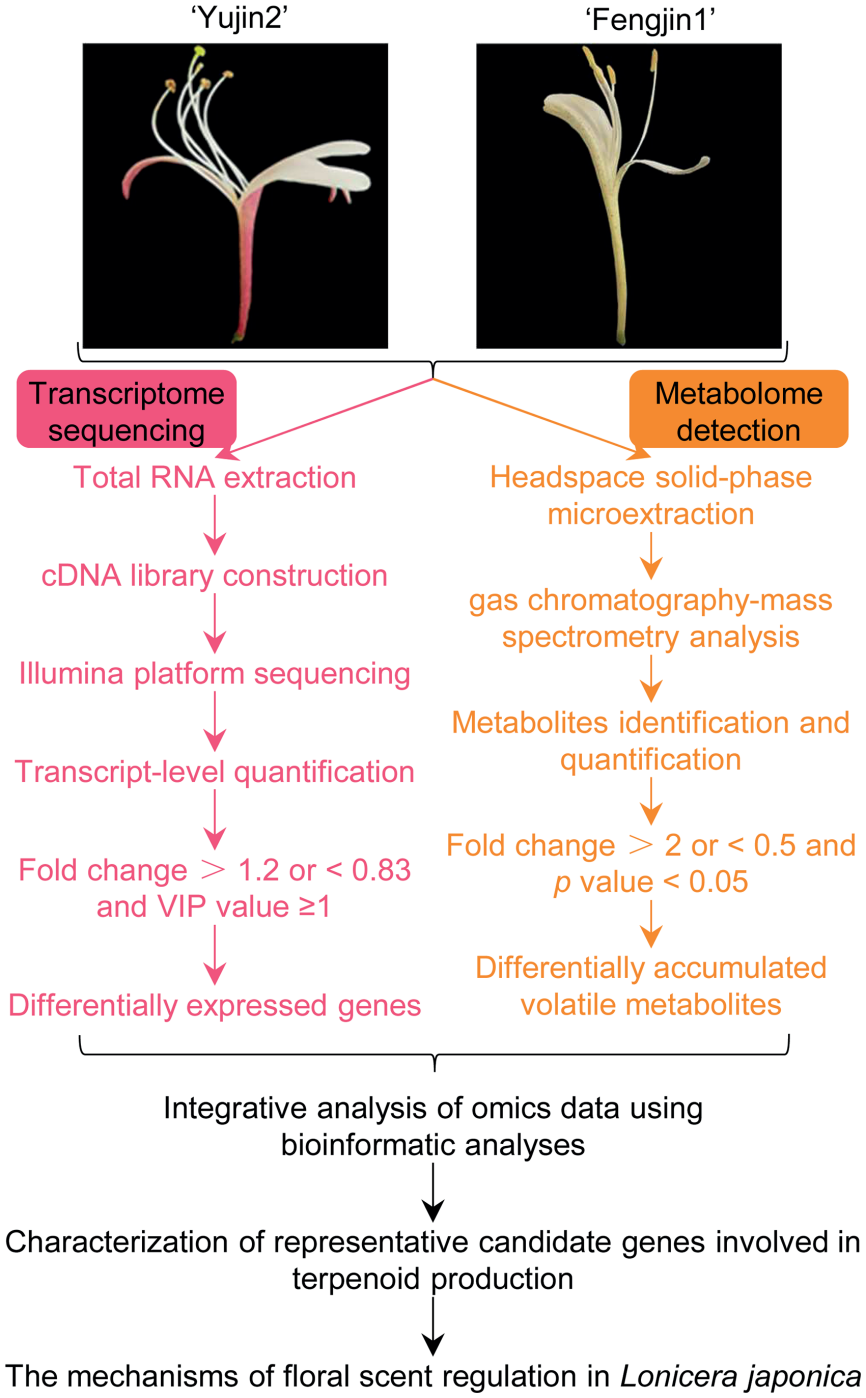
Overview of the metabolomics and transcriptomics workflow. *Lonicera japonica* flowers (LJFs) at the silver flowering stage of two contrasting varieties of *L. japonica,* ‘Yujin 2’ and ‘Fengjin 1’ were collected. Headspace solid-phase microextraction (HS-SPME) coupled with gas chromatography–mass spectrometry (GC–MS) and transcriptomics analysis were performed for volatile organic compound (VOC) profiling and global gene expression patterns, respectively. Integrated metabolomics and transcriptomics analysis serves to elucidate the regulatory mechanism of floral scents of LJFs by bioinformatic analyses.

In this study, headspace solid-phase microextraction (HS-SPME) coupled with GC–MS was performed for the identification of the VOCs of LJF at the silver stage (the stage with the strongest aroma) of these two contrasting varieties of *L. japonica* Thunb. Furthermore, correlation analysis of the transcriptomic profiles with metabolomic data was performed to elucidate the regulatory mechanism of the floral scents of LJF (Figure 1). Candidate genes were selected for validation *via* quantitative real-time PCR (qRT-PCR). These findings provide new information for the metabolic mechanisms and molecular basis of floral scents of LJF.

## Materials and methods

### Plant materials

LJFs at the silver flowering stage of two contrasting varieties of *L. japonica*, ‘Yujin 2’ and ‘Fengjin 1’, were collected in May 2019 in the Fengqiu honeysuckle germplasm resource nursery (Henan, China, 114°47′ N, 35°20′ E). Three biological replicates were performed, and each replicate was collected from at least three separate plants.

### HS-SPME and GC–MS analysis

The LJF samples were ground into powder in liquid nitrogen; then, 1.0 g of the powder was immediately placed in a 20 ml head-space vial containing 10 μl of internal standard (−)-carvone (50 μg/ml) and 2 ml of saturated NaCl solution. Then, at 100°C, the samples were shaken for 5 min, and a 120 μm divinylbenzene/carboxen/polydimethylsiloxane fiber was exposed to the headspace of the samples to absorb the volatiles for 15 min for GC–MS analysis.

The absorbed volatiles were analyzed using an Agilent Model 8,890 GC and a 5977B MS (Agilent Technologies, Stockport, United Kingdom) equipped with a DB-5MS capillary column (30 m × 0.25 mm × 0.25 μm). High-purity helium gas was used as a carrier gas with a velocity of 1.2 ml/min. The injector temperature was 250°C, and the detector temperature was 280°C. The oven temperature was programmed as follows: 40°C (3.5 min), increasing at 10°C/min to 100°C, at 7°C/min to 180°C, at 25°C/min to 280°C, and 280°C was maintained for 5 min. The MS was operated in full scan mode (50–500 amu at 1 scan/s) with electron ionization mode at 70 eV. The repeatability of the analysis process was monitored using a quality control (QC) sample after every 10 samples. Overlapping analysis of the total ion current in different QC samples was used to indicate the instrumental stability for GC–MS analysis.

Identification of VOCs was achieved by comparing the mass spectra with the data system library (MWGC) and linear retention index. Peak determination and peak area integration were carried out with MassHunter quantitative analyses (Agilent Technologies, Santa Clara, CA, United States). The relative content of each compound was calculated using the internal standard normalization method. Differentially accumulated volatiles (DAVs) were obtained using the threshold of quantitative fold change >1.2 or <0.83 and VIP value ≥1 between ‘Yujin2’ and ‘Fengjin1’. The odor qualities of the metabolites were obtained from the PubChem database,^1^ the Good Scent Company website,^2^ and the literature.

### RNA extraction, cDNA library construction and sequencing, transcript-level quantification, and DEG screening

Total RNA was isolated from 1 g of frozen sample using a mirVanaTM miRNA Isolation Kit (Ambion, Xian, China) according to the manufacturer’s protocol. The integrity and purity of the RNA were assessed using an Agilent 2,100 Bioanalyzer (Agilent Technologies, Santa Clara, CA, United States) and a NanoDrop microspectrophotometer (Thermo Fisher Scientific, Wilmington, DE, USA), respectively. The construction and sequencing of the cDNA library were conducted by OE Biotech Co., Ltd. (Shanghai, China). Three biological replicates per variety were conducted.

The clean reads were obtained by removing reads containing poly-N and low-quality reads from the raw data. Then, the clean reads were mapped to the *L. japonica* reference genome^3^ using HISAT. The read counts of each gene were obtained by htseq counting, and the fragments per kilobase million (FPKM) value of each gene was calculated using cufflinks. The differentially expressed genes (DEGs) were identified using the DESeq package in R software with a threshold of the fold change >2 or < 0.5 and *p* < 0.05.

### Bioinformatic analysis of omics data

Pearson’s correlation analysis and principal component analysis (PCA) were used to evaluate the data quality of the metabolome dataset and transcriptome dataset. Pearson’s correlation analyses were carried out in R software using the ‘corrplot’ package. PCA analyses were performed using the online OECloud tools.^4^ The hierarchical cluster analyses were performed using the R package ‘pheatmap’ with the “scale = row” parameter. Volcano plots were constructed using the online OmicShare platform.^5^

Identified metabolites were annotated using the Kyoto Encyclopedia of Genes and Genomes (KEGG) Compound database^6^ and mapped to the KEGG Pathway database.^7^ KEGG pathway enrichment analysis of DAVs was performed using Metabolite Set Enrichment Analysis (MSEA). The Gene Ontology (GO) and KEGG pathway enrichment analyses of DEGs were performed using R packages with hypergeometric distribution tests (Kanehisa et al., 2007). The DEGs and DAVs with Pearson’s correlation coefficient >0.8 and *p* < 0.05 were selected to construct the transcript-metabolite network, which was visualized by Cytoscape (version 3.7.1).

Transcription factors (TFs) were identified by searching against the Plant Transcription Factor Database (PlantTFDB 4.0),^8^ and the best hits with an E value less than 1e^−5^ were labeled as TFs. The specific target genes in Arabidopsis regulated by the TFs were predicted using the Gene Transcription Regulation Database (GTRD).^9^ The homologs of the predicted Arabidopsis target genes in *L. japonica* were acquired by sequence BLAST. To construct the TF regulatory network, the protein–protein interactions between TF target genes and the floral scent-related DEGs were analyzed using the STRING database (confidence score > 0.7).^10^ The TF regulatory network was visualized by Cytoscape (version 3.7.1).

Genome-wide identifications and phylogenetic analyses of the genes involved in pivotal VOCs production were performed. The Hidden Markov Models (HMMs) corresponding to the gene conserved domains were downloaded from the Pfam database^11^ and used as the query to identify the gene family from the *L. japonica* protein database^12^ using HMMER software. All the putative sequences were further confirmed to have complete conserved domains by the NCBI-CDD database.^13^ Phylogenetic analyses of the identified gene family members were performed using MEGAX 11.0 software with the maximum likelihood method.

### qRT-PCR analysis

The sequences of candidate genes were retrieved from the *L. japonica* genome database. Specific primer pairs used in qRT-PCR were designed using NCBI Primer-Blast^14^ (Supplementary Table 1). Total RNA of LJFs at the silver stage of ‘Yujin2’ and ‘Fengjin1’ was isolated using the OminiPlant RNA Kit (Cwbiotech, Beijing, China) and then reverse-transcribed using a HiScript® II Q RT SuperMix for qPCR kit (+ gDNA wiper; Vazyme Biotech, Nanjing, China). qRT-PCR was performed on a LightCycler® 96 real-time PCR system (Roche, Hong Kong, China) using SYBR Green Master Mix (Vazyme Biotech, Nanjing, China; Yu et al., 2021). Three biological and three technical replicates were performed. Relative expression levels were calculated using the 2^–ΔΔCt^ method (Liu et al., 2019). The *Lonja.ACT2/7* and *Lonja.27738* genes were used as internal references (Liu, 2017).

## Results

### Metabolome analysis of the LJFs of the two varieties of *Lonicera japonica*

**Figure 2:**
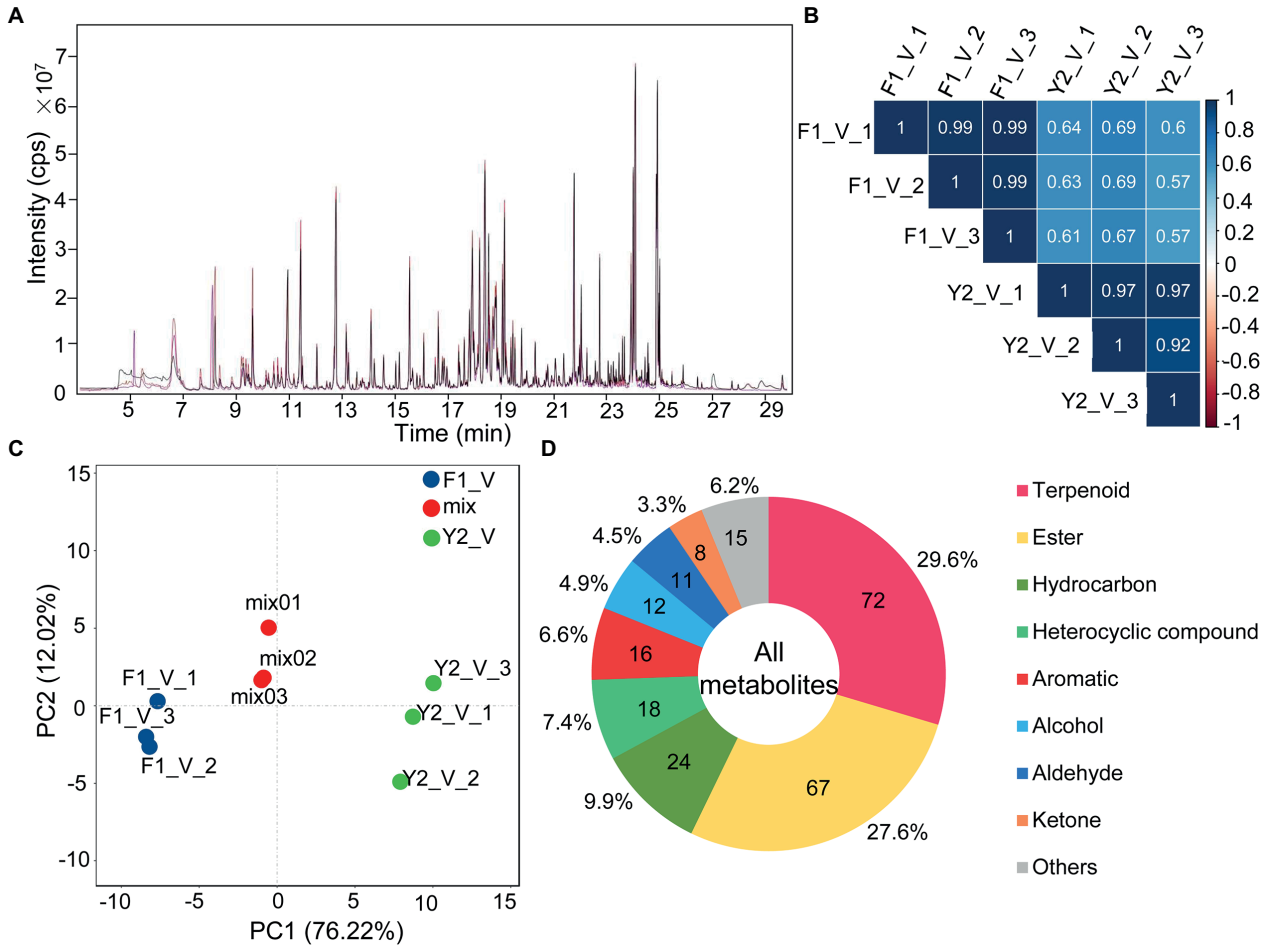
Overview of the metabolomics analysis. **(A)** Overlapping analysis of the total ion current in different quality control samples. The abscissa and the ordinate represent the retention time (min) and the intensity of the ion current (cps: count per second), respectively. **(B)** Pearson’s correlation analysis of LJF VOC profiles. **(C)** Principal component analysis (PCA) of LJF VOC profiles. **(D)** Component analysis of the identified VOCs of LJFs.

To understand what might contribute to the differences in the aroma of the LJFs of these two varieties, HS-SPME coupled with GC–MS was used to identify the VOCs of LJF at the silver stage (the stage with the strongest aroma) of ‘Yujin2’ and ‘Fengjin1’ (Figure 1). Overlapping analysis of the total ion current in different QC samples revealed the instrumental stability was good for GC–MS analysis (Figure 2A). Pearson’s correlation analysis showed weak correlations between ‘Yujin2’ and ‘Fengjin1’ samples, and strong correlations within the replicate samples (Figure 2B). PCA also showed that the VOC profiles of the two varieties were separate, and the biological replicates of the same variety were closely grouped (Figure 2C). These results suggest that the metabolome data were highly reproducible and reliable for further analyses, and the VOC profiles were different in the LJFs of the two varieties.

Despite distinct aroma characteristics between ‘Yujin2’ and ‘Fengjin1’, the identified metabolite species did not differ between the two varieties. In each of the variety, 243 VOCs were identified from LJFs (Supplementary Table 2). The detected VOCs were classified into different groups: 72 terpenoids (29.6%), 67 esters (27.6%), 24 hydrocarbons (9.9%), 18 heterocyclic compounds (7.4%), 16 aromatics (6.6%), 12 alcohols (4.9%), 11 aldehydes (4.5%), 8 ketones (3.3%), and 15 other compounds. Among these, terpenoids and esters accounted for 57.2% of the total VOCs (Figure 2D).

**Figure 3:**
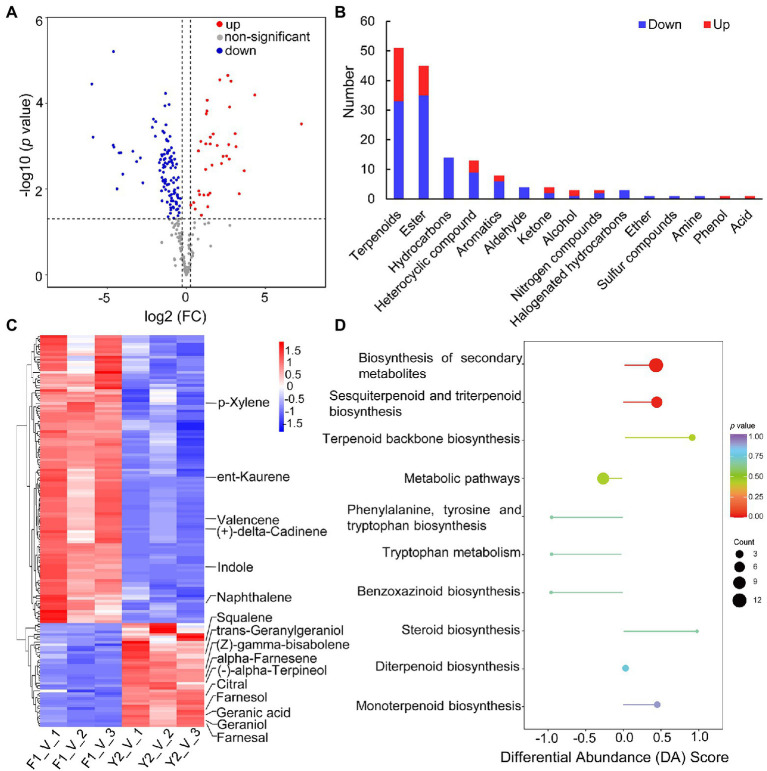
Characterization of differentially accumulated volatiles (DAVs) between ‘Yujin2’ and ‘Fengjin1’ LJF samples. **(A)** Volcano plot of the identified VOCs. The increased and decreased DAVs are shown in red and blue, respectively. **(B)** Abundance patterns of DAVs in each class. **(C)** Hierarchical clustering analysis of DAVs. The columns represent different samples, and the rows represent individual DAVs. The colors in the heat map represent the normalized values of DAVs, reflecting the relative contents. DAVs with high or low levels are indicated in red or blue, respectively. Detailed information is provided in Supplementary Table 4. The key DAVs that were functionally annotated through the Kyoto Encyclopedia of Genes and Genomes (KEGG) enrichment analysis are highlighted on the right. **(D)** KEGG enrichment analysis of the DAVs. The ordinate represents the enriched pathway name. The abscissa represents the differential abundance score (DA score), which reflects the overall change in DAVs in the pathway. The length of the line segment represents the absolute value of the DA score, and the size of the dot at the endpoint of the line segment represents the number of DAVs involved in the pathway. For dots shown on the right panel, the longer the line segment, the more inclined the overall expression of the pathway to be upregulated. In contrast, for dots shown on the left panel, the longer the line segment, the more inclined the overall expression of the pathway to be downregulated. The color of the line segment and the dot reflects the *p* value.

Interestingly, the levels of the VOCs from LJFs between the two contrasting varieties were quite different. A total of 153 DAVs were identified, of which 41 were increased and 112 were decreased in ‘Yujin2’ compared to ‘Fengjin1’ (Figure 3A; Supplementary Table 3). Most of the DAVs induced in ‘Yujin2’ were terpenoids and esters. However, the DAVs such as hydrocarbons, aldehydes, and halogenated hydrocarbons were decreased in ‘Yujin2’ (Figure 3B).

Notably, among these DAVs, 22 odorants were increased in ‘Yujin2’ compared to ‘Fengjin1’ (Table 1). These odorants have sweet, aromatic, floral, fruity, and other odor qualities. Most noteworthy, methyl anthranilate with a grape-like and orange blossom odor was increased 154.1-fold in ‘Yujin2’. Methyl anthranilate was ranked 15th among the identified VOCs in ‘Yujin2’ (Supplementary Table 2). The particularly high contents and extremely large increases of methyl anthranilate imply its crucial role for the strong aroma of ‘Yujin2’. Additionally, the accumulations of farnesol (delicate flowery odor), farnesal (floral minty aroma), trans-alpha-bergamotene (warm tea odor), squalene (faint agreeable odor), and ethyl cinnamate (sweet fruit odor) had more than 6-fold increases (Table 1). Among the increased odorants in ‘Yujin2’, in addition to methyl anthranilate, farnesol, alpha-farnesene (floral balsamic aroma), trans-alpha-bergamotene, and geraniol (sweet rose odor) were detected to be the top 20 ranking VOCs in ‘Yujin2’. The increased and high accumulation of these odorants may be the likely cause of the stronger aroma of LJFs in ‘Yujin2’ compared to that of ‘Fengjin1’.

**Table 1:** Odorants that were differentially accumulated in ‘Yujin2’ compared to ‘Fengjin1’.

Index	Common name	Class I	CAS	Fold change (Y2 vs. F1)	Odor quality
KMW0503	Methyl anthranilate	Ester	134-20-3	154.05	Grape-like odor; Orange blossom odor ⋆
NMW0310	Farnesol	Terpenoids	4602-84-0	12.64	Delicate flowery odor; Mild, oily; Weak citrus-lime odor ⋆
w05	Farnesal	Terpenoids	19317-114	8.48	Floral minty
KMW0555	trans-alpha-Bergamotene	Terpenoids	13474-59-4	7.09	Woody, warm tea
NMW0652	Squalene	Terpenoids	111-02-4	6.74	Faint agreeable odor ⋆
KMW0574	Ethyl cinnamate	Ester	103-36-6	6.67	Sweet, balsam, fruity, spicy, powdery, berry, plum
NMW0149	Geranic acid	Terpenoids	459-80-3	6.54	Dry, weedy, acidic, green, moldy feet, woody.
KMW0630	Nerolidol	Terpenoids	40716-66-3	5.86	A floral odor ⋆
KMW0514	Ethyl 3-phenylpropionate	Ester	2021-28-5	4.33	Hyacinth, rose, honey, fruity, rum
KMW0461	Nonanoic acid	Acid	112-05-0	2.86	Fatty odor; Coconut aroma; Slight odor ⋆
KMW0460	Geraniol	Terpenoids	106-24-1	2.85	A sweet rose odor; Pleasant geranium-like odor; Pleasant, floral odor ⋆
KMW0431	5-Methyl-6,7-dihydro-5H-cyclopenta[b]pyrazine	Heterocyclic compound	23747-48-0	2.56	Earthy, baked, potato, peanut, roasted
KMW0459	Citral	Terpenoids	141-27-5	2.49	Strong lemon odor ⋆
KMW0613	alpha-Farnesene	Terpenoids	502-61-4	2.46	A floral, green, and balsamic aroma ⋆
XMW0561	Farnesol, acetate	Ester	1000352-67-2	2.33	Delicate flowery odor; Mild, oily; Weak citrus-lime odor ⋆
KMW0716	Isopropyl palmitate	Ester	142-91-6	2.15	Almost odorless ⋆
XMW1084	cis-3-Hexenyl crotonate	Ester	65405-80-3	1.93	Green vegetable
XMW1486	guaiyl acetate	Ester	134-28-1	1.88	Tea, rose, woody, spicy, green fatty
NMW0071	(−)-alpha-Terpineol	Terpenoids	10482-56-1	1.73	Lilac floral terpenic
KMW0304	p-Mentha-1,3,8-triene	Terpenoids	18368-95-1	1.70	Turpentine camphor, herbal, woody
WMW0127	cis-3-Octen-1-ol	Alcohol	20125-84-2	1.44	Fresh fatty greasy, melon green cortex, herbal earthy fusel spicy
WMW0049	Nerylacetone	Ketone	3879-26-3	1.21	Fatty metallic

The information of the odor quality was obtained preferentially from the PubChem database (marked with pentagrams), and then from the Good Scent Company website.

To evaluate the accumulation patterns of the DAVs, hierarchical clustering analysis was performed, which revealed that the DAVs were grouped into two clusters (Figure 3C; Supplementary Table 4). Moreover, the DAVs were functionally annotated using the KEGG database to gain further insights into the mechanisms of the biosynthesis of DAVs (Figure 3D, Supplementary Table 5). The ‘Yujin2’-induced DAVs were predominantly enriched in the biosynthesis of secondary metabolites, especially terpenoid biosynthesis that included terpenoid backbone biosynthesis, monoterpenoid biosynthesis (alpha-terpineol and geraniol), diterpenoid biosynthesis (geranylgeraniol), and sesquiterpenoid and triterpenoid biosynthesis (farnesol, farnesal, alpha-farnesene, (Z)-gamma-bisabolene, and squalene). In contrast, indoles that were involved in phenylalanine, tyrosine, and tryptophan biosynthesis, as well as benzoxazinoid biosynthesis, were decreased in ‘Yujin2’ (Figure 3D).

### Transcriptome analysis of the LJFs of the two varieties of *Lonicera japonica*

**Figure 4:**
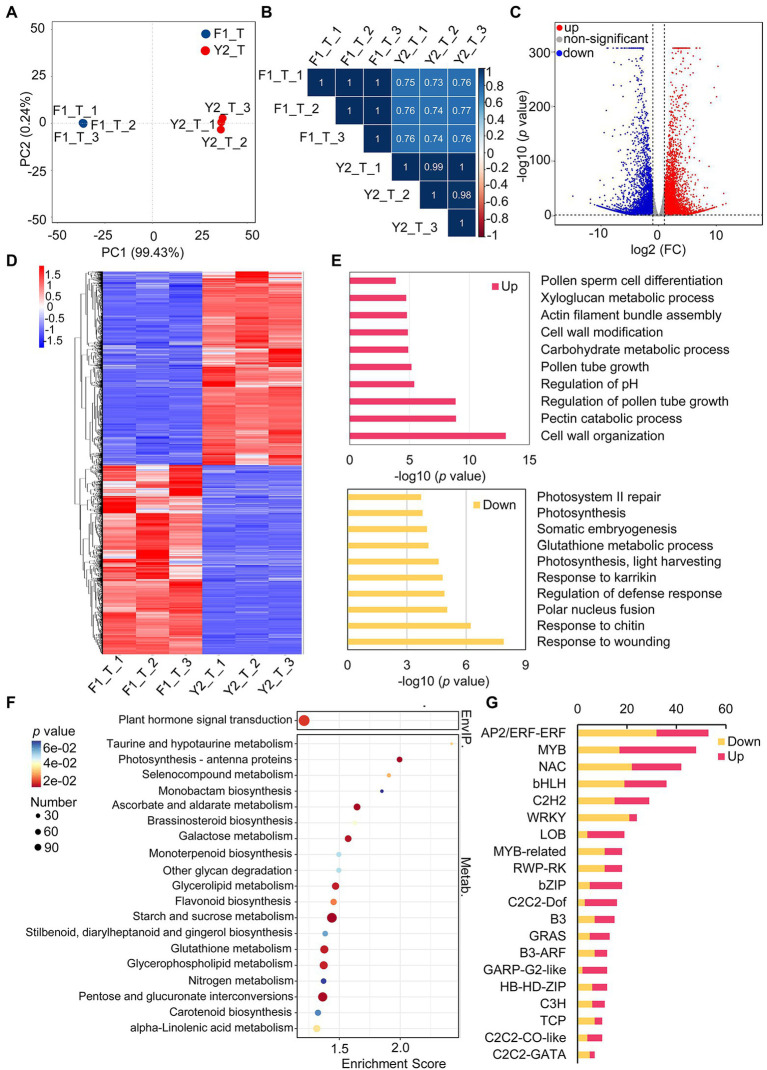
Characterization of differentially expressed genes (DEGs) between ‘Yujin2’ and ‘Fengjin1’ LJF samples. **(A)** PCA of the LJF transcriptome data. **(B)** Pearson’s correlation analysis of LJF transcriptome data. **(C)** Volcano plot of the identified DEGs. The increased and decreased DEGs are shown in red and blue, respectively. **(D)** Hierarchical clustering analysis of DEGs. The columns represent different samples, and the rows represent individual DEGs. The colors in the heat map represent the normalized values of DEGs. DEGs with high or low levels are indicated in red or blue, respectively. Detailed information is provided in Supplementary Table 9. **(E)** Gene Ontology biological process enrichment of DEGs. Detailed information is presented in Supplementary Table 10. **(F)** KEGG enrichment of DEGs. Detailed information is provided in Supplementary Table 11. **(G)** Top 20 differentially expressed transcription factor genes. Detailed information is provided in Supplementary Table 13.

In an attempt to obtain insights into the biosynthesis of the floral VOCs of the two varieties, transcriptomic analysis of the LJFs at the silver stage was performed (Figure 1). An average of 42 million clean reads per sample were aligned uniquely to the *L. japonica* genome (Supplementary Tables 6). PCA and Pearson’s correlation analysis revealed that the transcriptome data of LJF were reproducible of the same variety and different between the two varieties, which indicate the transcriptome data were reliable for further analyses (Figures 4A, B).

A total of 25,649 and 25,943 expressed genes were quantified in the LJF samples from ‘Yujin2’ and ‘Fengjin1’, respectively (Supplementary Table 7). A total of 9,523 DEGs were identified, of which 4,715 genes were upregulated and 4,808 genes were downregulated in ‘Yujin2’ compared to ‘Fengjin1’ (Figure 4C; Supplementary Table 8). Hierarchical clustering analysis of the DEGs was performed, and the DEGs were grouped into two clusters (Figure 4D; Supplementary Table 9).

Based on this clustering, the DEGs were functionally annotated using the GO and KEGG enrichment analyses. According to the biological process, the upregulated DEGs were mainly involved in cell wall organization, pectin catabolic process, regulation of pollen tube growth, regulation of pH, carbohydrate metabolic process, etc. With regard to downregulated DEGs, most of them were enriched in the regulation of defense response (including the response to wounding, chitin, and karrikin), polar nucleus fusion, photosynthesis, glutathione metabolic process, and so on (Figure 4E; Supplementary Table 10). Moreover, KEGG pathway enrichment analysis revealed that the DEGs were assigned to 127 KEGG pathways (Supplementary Table 11). The most significantly enriched KEGG pathways are shown in Figure 4F. Notably, brassinosteroid biosynthesis, monoterpenoid biosynthesis, and carotenoid biosynthesis, which are related to the metabolism of terpenoids and polyketides, were significantly enriched (Figure 4F). In addition to these, other pathways related to terpenoids and polyketides were also enriched, including terpenoid backbone biosynthesis, sesquiterpenoid and triterpenoid biosynthesis, as well as diterpenoid biosynthesis. Furthermore, additional pathways associated with VOC biosynthesis were also enriched, including steroid biosynthesis, phenylalanine, tyrosine, and tryptophan biosynthesis, phenylpropanoid biosynthesis, as well as alpha-linolenic acid metabolism (Supplementary Table 12).

**Figure 5:**
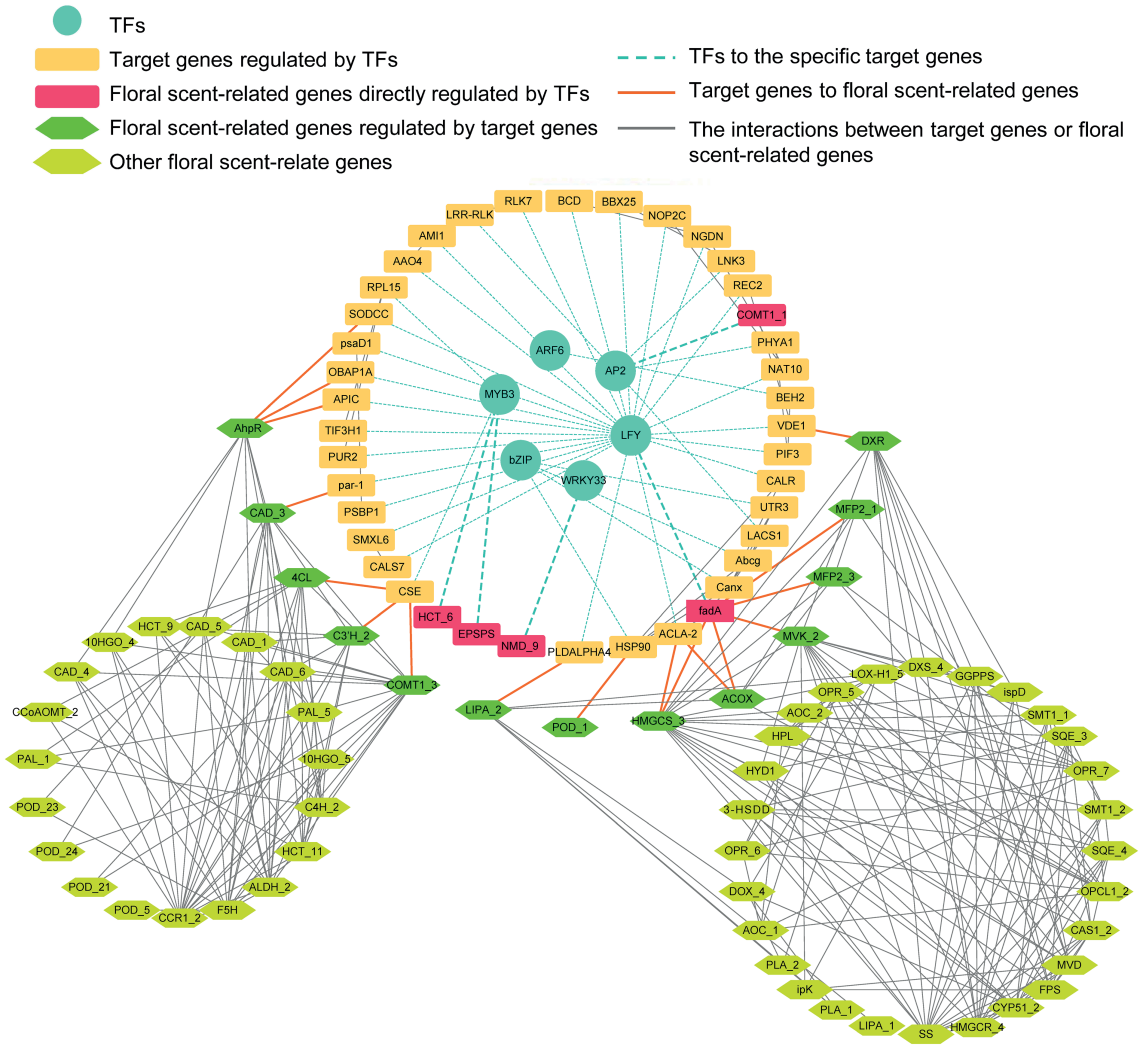
A regulatory network of transcription factors (TFs) and the floral scent-related genes. The circular, rectangular, hexagonal nodes indicate the TFs, target genes, and floral scent-related genes, respectively. The turquoise, orange, and gray edges indicate the TFs to the specific target genes predicted by GTRD, target genes to floral scent-related genes and the interactions between floral scent-related genes predicted by STRING. The raw data and the full names of the abbreviations can be found in Supplementary Table 15.

Notably, a total of 520 TFs distributed among 56 TF families were differentially expressed in ‘Yujin2’ compared to ‘Fengjin1’. Among these, almost half of the differentially expressed TFs are classified into six families, i.e., APETALA2/ethylene-responsive factor (AP2/ERF-ERF), v-myb avian myeloblastosis viral oncogene homolog (MYB), NAC, WRKY, Basic helix–loop–helix (bHLH), and C2H2. Interestingly, most of the TFs in the GARP-G2-like family and the lateral organ boundary domain (LOB) family were upregulated in ‘Yujin2’, while the TFs in the WRKY family were mostly downregulated (Figure 4G; Supplementary Table 13). To further investigate the TFs related to VOC production in floral scent of LJF, these differentially expressed TFs were used to construct a regulatory network of TFs and the floral scent-related genes (Figure 5, Supplementary Table 12 and 13). Six TF families, including MYB3, AP2, LFY, ARF6, WRKY33 and bZIP TFs, were predicted to regulate 40 target genes that were directly or indirectly associated with the floral scent-related genes. Among the 40 target genes, 25 were predicted to be regulated by LFY, which was increased 5.81-fold in ‘Yujin2’. This indicates that LFY was an important TF in the regulatory network of floral scent in LJF. Among the LFY-regulated target genes, acetyl-CoA acyltransferase (fadA) that involved in alpha-linolenic acid metabolism was increased 3.3-fold in ‘Yujin2’ and interacted with several floral scent-related genes, such as mevalonate kinase (MVK) and hydroxymethylglutaryl-CoA synthase (HMGCS) that involved in terpenoid biosynthesis, as well as acyl-CoA oxidase (ACOX) and fatty acid beta-oxidation multifunctional protein (MFP2) that involved in alpha-linolenic acid metabolism. This suggests the important role of fadA in the regulatory network. In addition, a caffeate O-methyltransferase (COMT1) and a hydroxycinnamoyl transferase (HCT), which were upregulated in ‘Yujin2’, were predicted to be regulated by AP2 and MYB3, respectively. A (+)-neomenthol dehydrogenase (NMD) and a 5-enolpyruvylshikimate 3-phosphate synthase (EPSPS), which were downregulated in ‘Yujin2’, were predicted to be regulated by WRKY33 and MYB3, respectively. Moreover, there are a number of genes related to floral scent that were predicted to be directly or indirectly regulated by the TF-target genes, suggesting they are likely to be indirectly regulated by the TFs.

### Integrated volatile metabolome and transcriptome analysis of VOC accumulation in LJFs

**Figure 6:**
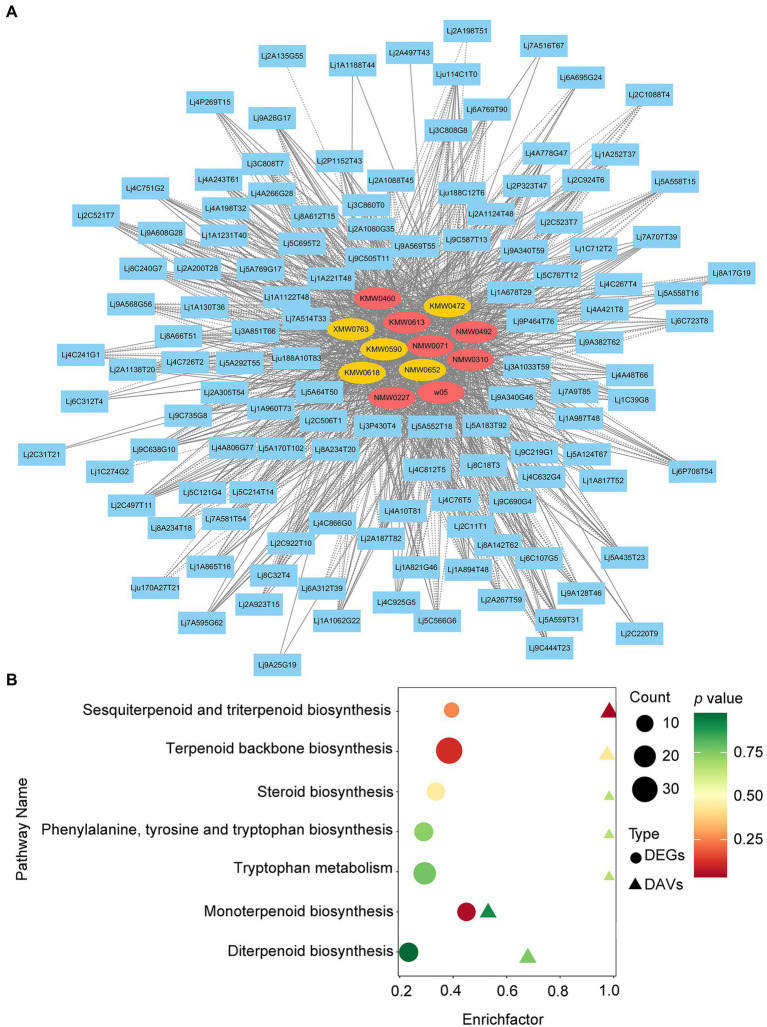
Integrated volatile metabolome and transcriptome analysis of VOC accumulation in LJFs. **(A)** Gene-metabolite correlation network representing DEGs and DAVs. The gene-metabolite pairs are connected by edges. Blue rectangular nodes represent genes. Red and yellow elliptical nodes represent increased and decreased metabolites, respectively. Solid and dashed edges represent positive and negative correlations, respectively. **(B)** Bubble map of KEGG pathways co-enriched from metabolome and transcriptome data. The ordinate represents KEGG terms, and the abscissa represents the enrichment factor of each term.

To generate the candidate genes likely to be involved in LJF VOC biosynthesis, we integrated the transcriptome data and the VOC profiling data by correlation analysis (Figure 6A; Supplementary Table 14). In total, 12 DAVs showed higher corrections with DEGs, which included geraniol, L-alpha-terpineol, alpha-farnesene, and trans-geranylgeraniol, etc. Based on KEGG analysis, the DEGs and DAVs between ‘Yujin2’ and ‘Fengjin1’ were integrated into several pathways related to VOC biosynthesis, including sesquiterpenoid and triterpenoid biosynthesis, terpenoid backbone biosynthesis, steroid biosynthesis, phenylalanine, tyrosine and tryptophan biosynthesis, tryptophan metabolism, monoterpenoid biosynthesis, and diterpenoid biosynthesis (Figure 6B).

### Genome-wide identification and phylogenetic analyses of the genes involved in pivotal VOCs production in *Lonicera japonica*

To gain further insight into the evolutionary relationships and expression patterns among the subfamilies of the genes involved in pivotal VOCs production, phylogenetic trees of the gene families were constructed (Supplementary Figure 1). The phylogenetic analyses of multiple gene family members that were differentially regulated in ‘Yujin2’ revealed the gene expression of the members associated with the evolution of the gene family, including TPS, TIDS, 1-deoxy-D-xylulose-5-phosphate synthase (DXS), HMGCS, hydroxymethylglutaryl-CoA reductase (HMGCR), MVK, squalene epoxidase 1 (SQE1), and LUS. For instance, three DXS members of the clade I were upregulated, while another DXS member of the clade II was downregulated. Similar change patterns in different clades of the subfamilies of the gene family were also observed in HMGCS, HMGCR, MVK, LUS, and SQE1. The TPS gene family was divided into seven subfamilies a-g as previously reported (Supplementary Figure 2; Jiang et al., 2019). The differentially regulated family members of alpha-farnesene synthase 1 (AFS1) and TES both belong to TPS-b subfamilies but in different clades. These results suggest the members of these gene families play different roles in terpenoid biosynthesis.

### Characterization of terpenoid biosynthesis-related genes in LJFs

**Figure 7:**
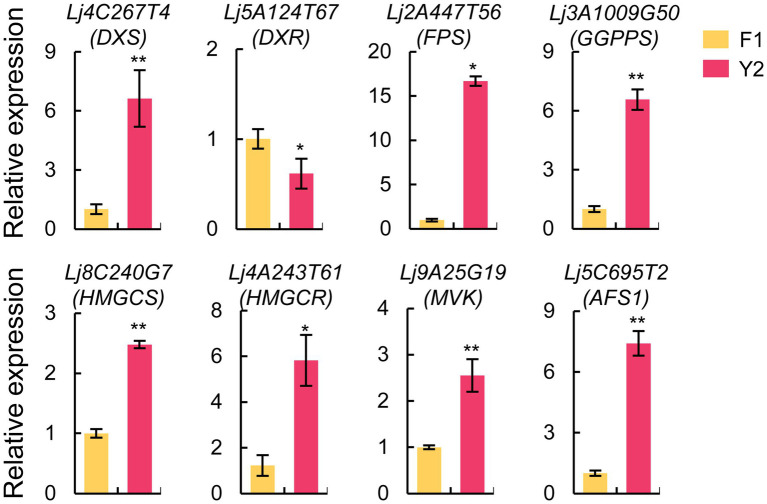
Expression analysis of candidate genes involved in the terpenoid biosynthetic pathway based on quantitative real-time PCR (qRT-PCR). The actin gene was used as an internal reference. The values are presented as the mean ± standard deviation (*n* = 3). Significant differences between ‘Yujin2’ and ‘Fengjin1’ are marked with asterisks (**p < 0.01, *p < 0.05, Student’s *t*-test). AFS1, alpha-farnesene synthase; DXR, 1-deoxy-D-xylulose-5-phosphate reductoisomerase; DXS, 1-deoxy-D-xylulose-5-phosphate synthase; FPS, farnesyl diphosphate synthase; GGPPS, geranylgeranyl diphosphate synthase; HMGCR, hydroxymethylglutaryl-CoA reductase; HMGCS, hydroxymethylglutaryl-CoA synthase; MVK, mevalonate kinase.

The expression patterns of the candidate genes involved in terpenoid biosynthesis were verified using qRT-PCR (Figure 7; Supplementary Figure 3). The results showed that seven genes were upregulated, while one gene was downregulated in ‘Yujin2’ compared to ‘Fengjin1’, similar to the trends revealed by transcriptomic analysis. These verified genes included DXS, 1-deoxy-D-xylulose-5-phosphate reductoisomerase (DXR), and geranylgeranyl diphosphate synthase (GGPPS), involved in the 2-c-methylerythritol 4-phosphate (MEP) pathway, and four genes involved in the mevalonic-acid (MVA) pathway, including HMGCS, HMGCR, MVK, and AFS1.

## Discussion

The regulatory mechanisms of floral scents are sophisticated and complex. Integrated metabolomic and transcriptomic analysis allows for representation of gene-to-metabolite networks to decipher the mechanisms involved in the regulation of LJF floral scents. In the current study, the metabolomic and transcriptomic analyses of LJFs at the silver flowering stage of ‘Yujin2’ and ‘Fengjin1’ indicated that the biosynthesis of terpenoids and amino acid and fatty acid derivatives was pivotal for the stronger aroma in ‘Yujin2’ compared to ‘Fengjin1’.

### Differential accumulation of key odorants contributes to the stronger aroma of LJFs in ‘Yujin2’

Previous studies have found that the primary VOCs of LJFs are monoterpenes, sesquiterpenes, fatty acids, and their esters (Wang et al., 2009; Ilie et al., 2014). With very few exceptions, the VOCs of LJFs reported in previous studies were also detected in this study (Supplementary Table 2; Wang et al., 2009; Ilie et al., 2014; Du et al., 2015; Lin et al., 2020; Yan et al., 2020). Floral scents tend to be mixtures of many compounds, but there are always major compounds that contribute the most significantly. In line with previous studies, the characteristic aromatic compounds of LJFs were also detected at high levels in the two varieties in this study, such as terpenoids that included linalool, farnesol, alpha-farnesene, delta-cadinene, trans-alpha-bergamotene, and geraniol, as well as esters that included methyl anthranilate and geranyl isovalerate (Supplementary Table 2; Wang et al., 2009; Ilie et al., 2014; Du et al., 2015; Lin et al., 2020; Yan et al., 2020). Among these abundant VOCs, linalool and geranyl isovalerate exhibited no significant difference between ‘Yujin2’ and ‘Fengjin1’. Interestingly, the abundant VOCs that included methyl anthranilate, farnesol, alpha-farnesene, trans-alpha-bergamotene, and geraniol, as well as other VOCs that included squalene, nerolidol, hedycaryol, nonanoic acid, citral, isopropyl palmitate, and alpha-terpineol, accumulated more in ‘Yujin2’ compared to ‘Fengjin1’ (Table 1). These increased odorants, which have sweet, pleasant, floral, and fruity odor qualities, are likely to be the reasons for the stronger aroma of LJFs in ‘Yujin2’ compared to ‘Fengjin1’. Notably, methyl anthranilate, a grape scent and flavor compound, can impart a pleasant aroma (Luo et al., 2019). In consideration of its most significant increase and high accumulation, methyl anthranilate would likely be the greatest cause of the stronger aroma in ‘Yujin2’ compared to ‘Fengjin1’.

It is important for plants to emit floral scents in order to attract pollinators, deter pathogens, and respond to biotic and abiotic stressors (Schiestl, 2015). Linalool and E-β-ocimene are reported as being attractive to pollinators including bees and moths to enhance propagation (Parachnowitsch et al., 2012; Gerard et al., 2017). No significant difference in the accumulation of linalool and E-β-ocimene between ‘Yujin2’ and ‘Fengjin1’ suggested that the ability of these two varieties to attract pollinators was not significantly different, despite differences in the LJF aroma (Supplementary Table 2). (E)-α-bergamotene and particular homoterpenes or terpene derivatives help to protect plants from microbial pathogens and abiotic stresses by recruiting the enemies of pests, and (E, E)-α-farnesene has been reported to influence multiple interactions between plants and other organisms when exposed to abiotic stresses (Zhou et al., 2017). The significantly increased α-farnesene and α-bergamotene in ‘Yujin2’ may contribute to its high resistance to pathogens, as reported previously (Li et al., 2016).

### Terpenoid biosynthetic pathway was promoted in ‘Yujin2’ leading to the accumulation of terpenoids

**Figure 8:**
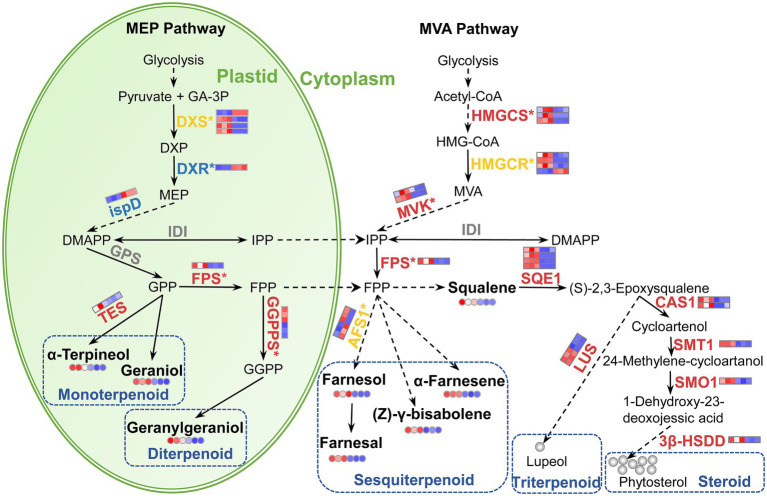
Schematic presentation of terpenoid biosynthetic pathway in LJFs of ‘Yujin2’ compared to ‘Fengjin1’. The upregulated, downregulated, mix-regulated, and unchanged genes (bold fonts) and metabolites (small circles) in ‘Yujin2’ are represented in red, blue, yellow, and gray, respectively. The scale bars adjacent to the name of genes or metabolites are picked from the heatmaps for the transcriptome (Figure 4D) and metabolome (Figure 3C). The first three and the latter three squares of a bar represent the normalized abundance values of DEGs or DAVs from ‘Yujin2’ and ‘Fengjin1’, respectively. Red and blue colors reflect high and low expression levels, respectively. The asterisk followed by the gene name indicate that the gene expression was validated by qRT-PCR. The solid line, dashed line, and dotted line indicate a single-step reaction, a multi-step reaction, and the movement of substances, respectively. 3β-HSDD, plant 3β-hydroxysteroid-4α-carboxylate 3-dehydrogenase; Acetyl-CoA, acetoacetyl-CoA; AFS1, alpha-farnesene synthase; CAS1, cycloartenol synthase; DMAPP, dimethylallyl pyrophosphate; DXP, 1-deoxy-d-xylulose 5-phosphate; DXR, DXP reductoisomerase; DXS, DXP synthase; FPP, farnesyl diphosphate; FPS, farnesyl diphosphate synthase; GA-3P, D-glyceraldehyde 3-phosphate; GGPP, geranylgeranyl diphosphate (C20); GGPPS, GGPP synthase; GPP, geranyl diphosphate; GPS, geranyl diphosphate synthase; HMG-CoA, 3-hydroxy-3-methylglutaryl-CoA; HMGR, HMG-CoA reductase; HMGS, HMG-CoA synthase; IDI, IPP isomerase; IPP, isopentenyl diphosphate; LUS, lupeol synthase; MEP, methylerythritol phosphate; MVA, mevalonic acid; MVK, mevalonate kinase; SMO1, plant 4,4-dimethylsterol C-4alpha-methyl-monooxygenase; SMT1, sterol 24-C-methyltransferase; SQE1, squalene monooxygenase; TPS, alpha-terpineol synthase.

Terpenoids are the most dominant and diverse group of floral VOCs (Muhlemann et al., 2014). In the current study, terpenoids were the largest group identified in JLFs (Figure 2D). In higher plants, terpenoids are derived from the C5 carbon precursors isopentenyl diphosphate (IPP) or dimethylallyl diphosphate (DMAPP) through two alternative pathways, the plastid-localized MEP pathway and the cytosol-localized MVA pathway in flowers and other plant organs (Figure 8; Dudareva et al., 2013).

Monoterpenes and diterpenes represent important classes of aromatic compounds responsible for floral scents (Qiao et al., 2021). Increased accumulations of geraniol (sweet rose odor) and alpha-terpineol (lilac floral terpenic odor), i.e., monoterpenes, and geranylgeraniol, i.e., a diterpene, in ‘Yujin2’, may contribute to the stronger aroma (Figure 8). Monoterpenes and diterpenes are generated by the plastid-localized MEP pathway. Beginning with pyruvate and glyceraldehyde-3-phosphate, DXS, DXR, and 2-C-methyl-D-erythritol 4-phosphate cytidylyltransferase (ispD) catalyze the first three reactions of the MEP pathway (Qiao et al., 2021). In this study, we found three DXS genes that were upregulated, while a DXS gene, a DXR, and an ispD were downregulated in ‘Yujin2’ (Figure 8; Supplementary Table 12). These results suggested that the biosynthesis of the C5 carbon precursors IPP and DMAPP *via* the MEP pathway may be inhibited in ‘Yujin2’. However, GGPPS, which primarily utilizes the products of the MEP pathway to condense three IPP molecules and one DMAPP molecule into geranylgeranyl diphosphate (GGPP), was upregulated in ‘Yujin2’ (Figure 8; Supplementary Table 12). GGPP serves as the entry point leading to the biosynthesis of a diverse group of primary and secondary terpenoid compounds, including monoterpenes, diterpenoids, and plastid-derived carotenoids and their derivatives (Nagegowda and Gupta, 2020). It has been widely reported that GGPPS contributes to the production of monoterpenes in flowering plants, such as *Arabidopsis thaliana* (Chen et al., 2015), *Chimonanthus praecox* (Shang et al., 2020), *Thymus caespititius* (Adal and Mahmoud, 2020), *Antirrhinum majus*, and *Clarkia breweri* (Tholl et al., 2004). Additionally, we found that several genes involved in monoterpenoid biosynthesis were differentially regulated in ‘Yujin2’, including increased alpha-terpineol synthase (TES) and mix-regulated 8-hydroxygeraniol dehydrogenase (10HGO) and NMD (Figure 8; Supplementary Table 12). TES catalyzes the conversion of geranyl diphosphate (GPP) to alpha-terpineol (Lima et al., 2013). Our results indicated that the upregulation of GGPPS and TES plays crucial roles in increasing the accumulation of geraniol, alpha-terpineol, and geranylgeraniol in ‘Yujin2’.

Sesquiterpenoids have also been identified as important components of floral scents. In this study, three odorants classified as sesquiterpenoids increased significantly in ‘Yujin2’, including farnesol (weak citrus-lime odor), farnesal (white-lemon-like aroma), and alpha-farnesene (floral, green, and balsamic aroma; Table 1; Supplementary Table 3). Sesquiterpenoids are synthesized *via* the MVA pathway in the cytosol. In the MVA pathway, three genes of HMGCS, three genes of HMGCR, as well as two genes of MVK, which catalyze reactions to produce IPP and DMAPP from the condensation of acetyl-CoA, were upregulated significantly in ‘Yujin2’ (Figure 8; Supplementary Table 12). This implied that the MVA pathway producing the C5 carbon precursors IPP and DMAPP in the cytosol was promoted in ‘Yujin2’. Consistently, farnesyl pyrophosphate synthase (FPS) was also upregulated. FPS condenses two IPP molecules and one DMAPP molecule to produce farnesyl diphosphate, which can be converted to sesquiterpenoids catalyzed by cytosolic sesquiterpene synthases (Chen et al., 2011). The significantly upregulated HMGCS, HMGCR, MVK, and FPS in ‘Yujin2’ were closely related to the increases in sesquiterpenoids responsible for the strong aroma.

Triterpenoids are often reported as components of the floral scent in LJFs (Wang et al., 2016; Li et al., 2020). Triterpenoids are biosynthesized *via* different cyclization reactions of squalene. In the current study, squalene with faint agreeable odor was increased significantly in ‘Yujin2’ (Table 1). In agreement with this, we also found four genes of SQE1, which oxidizes squalene to squalene 2,3-epoxide, and two genes of lupeol synthase (LUS), which converts oxidosqualene to other triterpene alcohols, that were significantly upregulated in ‘Yujin2’ (Figure 8; Supplementary Table 12). In addition, squalene is a precursor for the synthesis of plant sterols. We also found that four genes involved in catalyzing squalene 2,3-epoxide to form phytosterols were significantly upregulated, including two genes of cycloartenol synthase (CAS1), sterol 24-C-methyltransferase (SMT1), 4,4-dimethylsterol C-4alpha-methyl-monooxygenase (SMO1), and plant 3-beta-hydroxysteroid-4-alpha-carboxylate 3-dehydrogenase (3β-HSDD; Supplementary Table 12). Our results revealed that the biosynthesis of triterpenoids and phytosterols was induced in ‘Yujin2’.

### Phenylpropanoids and benzenoids did not contribute to the stronger aroma of ‘Yujin2’

**Figure 9:**
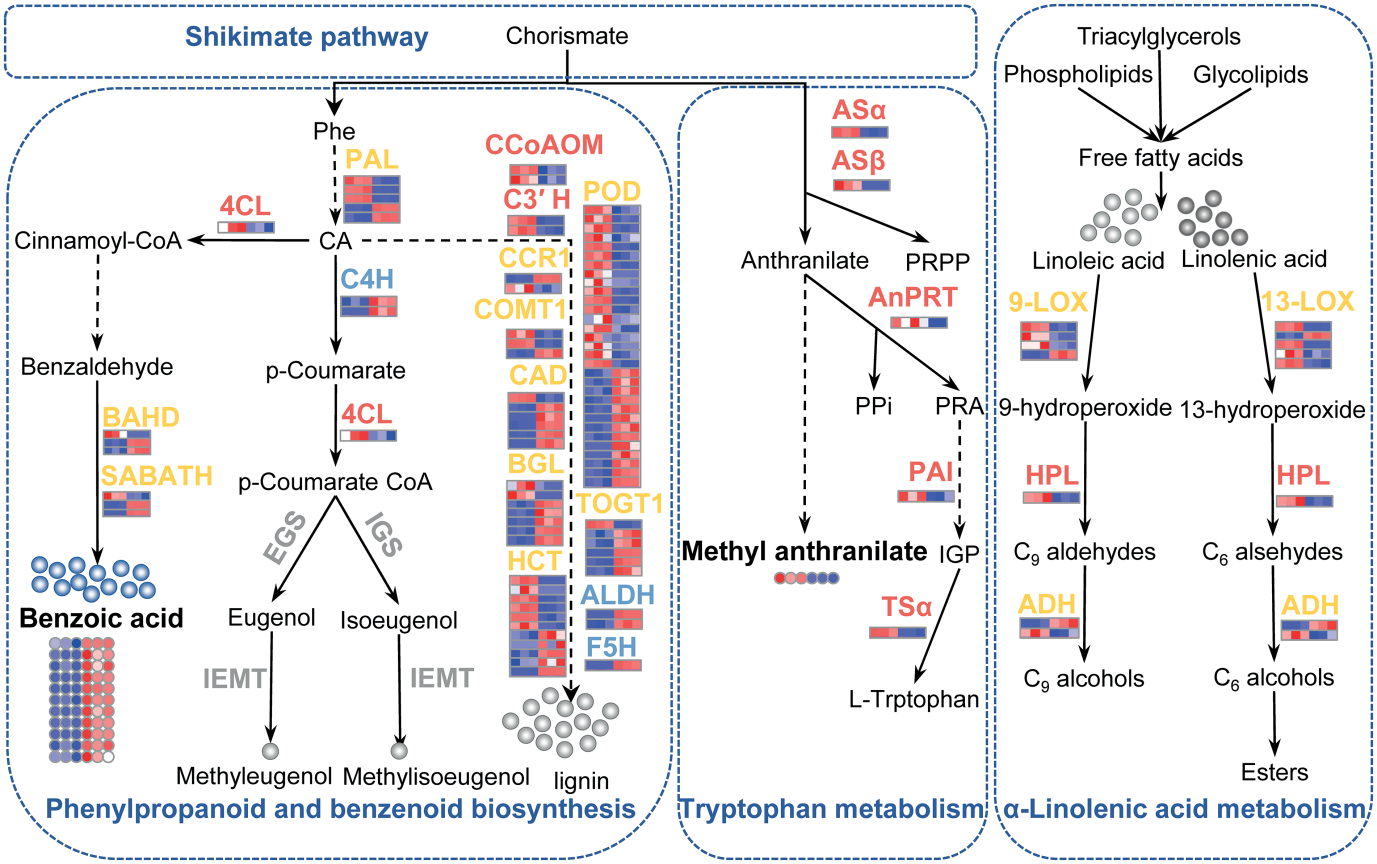
Schematic presentation of the biosynthesis of phenylpropanoids, benzenoids, and tryptophan, as well as alpha-linolenic acid metabolism in LJFs of ‘Yujin2’ compared to ‘Fengjin1’. The upregulated, downregulated, mix-regulated, and unchanged genes (bold fonts) and metabolites (small circles) in ‘Yujin2’ are represented in red, blue, yellow, and gray, respectively. The scale bars adjacent to the name of genes o are picked up from the heatmaps for the transcriptome (Figure 4D). The first three and the latter three squares of a bar represent the normalized abundance values of DEGs from ‘Yujin2’ and ‘Fengjin1’, respectively. Red and blue colors reflect high and low expression levels, respectively. The asterisk followed by the gene names indicate that gene expression was validated by qRT-PCR. The solid line and dashed line indicate single-step reactions and multi-step reactions, respectively. 4CL, 4-hydroxycinnamoyl CoA ligase; ADH, alcohol dehydrogenase; ALDH, coniferyl-aldehyde dehydrogenase; AnPRT, anthranilate phosphoribosylanthranilate transferase; ASα, ASβ, anthranilate synthase; BGL, beta-glucosidase; C3′ H, 5-O-(4-coumaroyl)-D-quinate 3′-monooxygenase; C4H, cinnamate 4-hydroxylase; CA, trans-cinnamate; CAD, cinnamyl alcohol dehydrogenase; CCoAOM, caffeoyl-CoA O-methyltransferase; CCR1, cinnamoyl-CoA reductase; COMT1, caffeic acid 3-O-methyltransferase; EGS, eugenol synthase; F5H, ferulate-5-hydroxylase; HCT, shikimate O-hydroxycinnamoyl transferase; HPL, hydroperoxide lyase; IEMT, isoeugenol O-methyltransferase; IGS, isoeugenol synthase; LOX, lipoxygenase; PAI, phosphoribosylanthranilate isomerase; PAL, phenylalanine ammonia-lyase; Phe, phenylalanine; POD, peroxidase; PPi, inorganic pyrophosphate; PRPP, phosphoribosyl pyrophosphate; SABATH, S-adenosylmethionine-dependent methyltransferase; TOGT1, scopoletin glucosyltransferase; TSα, tryptophan synthase alpha subunit.

Phenylpropanoids and benzenoids are the second most ubiquitous class of plant VOCs (Knudsen and Gershenzon, 2006). Most VOCs from this class are derived from L-phenylalanine *via* the shikimate pathway. The first step in the biosynthesis of most phenylpropanoids and benzenoids is the deamination of L-Phe to trans-cinnamic acid (CA) by the enzyme L-Phe ammonia-lyase (PAL; Dudareva and Pichersky, 2006). Among the identified PAL genes, three were upregulated and two were downregulated in ‘Yujin2’ (Figure 9; Supplementary Table 12). Several genes involved in phenylpropanoid biosynthesis were upregulated in ‘Yujin2’, including 4-coumarate-CoA ligase-like 1 (4CL), p-coumaroyl quinate/shikimate 3′-hydroxylase (C3′H), and caffeoyl-CoA O-methyltransferase (CCoAOM). There were also a large number of genes related to phenylpropanoid biosynthesis that were downregulated or mix-regulated in ‘Yujin2’. However, a series of enzymes required for biosynthesis of the volatiles methyleugenol and methyl isoeugenol were not differentially expressed between ‘Yujin2’ and ‘Fengjin1’, including coniferyl alcohol acyltransferase that produces coniferyl acetate, eugenol synthase and isoeugenol synthase that convert coniferyl acetate to eugenol and isoeugenol, respectively, as well as isoeugenol O-methyltransferase required for methylation to produce the volatiles methyleugenol and methyl isoeugenol (Dudareva et al., 2013; Mostafa et al., 2022). Consistent with the lack of significant expression differences of methyleugenol and methyl isoeugenol-related genes, differences in volatile phenylpropanoid accumulation between ‘Yujin2’ and ‘Fengjin1’ were not detected in the volatile profiling of LJFs (Figure 9; Supplementary Table 3). These results suggest that the differentially regulated genes involved in the phenylpropanoid biosynthesis pathway mainly contribute to the biosynthesis of non-volatile metabolites such as lignin in LJFs, rather than the biosynthesis of volatile phenylpropanoids (Peled-Zehavi et al., 2015). Our results also indicate that the differences in the floral scents between ‘Yujin2’ and ‘Fengjin1’ have a weak relationship with volatile phenylpropanoid accumulation.

Notably, all of the 11 detected volatile benzenoids were decreased in ‘Yujin2’, i.e., benzaldehyde, eight ester derivatives of benzoic acid (benzyl salicylate, benzyl tiglate, hexyl benzoate, methyl benzoate, ethyl salicylate, benzyl benzoate, 3-hexen-1-ol benzoate, and 3-hexen-1-ol, benzoate, (z)-), as well as two other benzene-substituted derivatives—benzyl chloride and ethyl phenylacetate (Supplementary Table 3). Benzenoids are synthesized from phenylalanine *via* the benzenoid branch of the phenylpropanoid pathway. Consistent with the decreased volatile benzenoids, two genes of BAHD acyltransferase and two genes of SABATH methyltransferase, involved in the final steps of benzenoid volatile formation, were significantly downregulated in ‘Yujin2’ (Figure 9; Supplementary Table 12; D’Auria et al., 2003; D’Auria, 2006). These results indicated that the biosynthesis and accumulation of benzenoids were suppressed, which were assumed to not contribute to the stronger aroma of ‘Yujin2’.

### Tryptophan biosynthetic pathway was elevated in ‘Yujin2’

In addition to phenylalanine acting as a precursor for phenylpropanoids and benzenoids, two other aromatic amino acids (tyrosine and tryptophan) are precursors for various plant aromatic secondary metabolites (Tzin and Galili, 2010). In the biosynthesis of these three aromatic amino acids, chorismate is the common intermediate. Specifically, a series of genes involved in producing tryptophan from chorismate were upregulated in ‘Yujin2’, including the anthranilate synthase alpha subunit (ASα) and beta subunit (ASβ), anthranilate phosphoribosyltransferase (AnPRT), N-(5′-phosphoribosyl) anthranilate isomerase 1 (PAI1), and tryptophan synthase alpha chain (TSα; Figure 9; Supplementary Table 12; Tzin and Galili, 2010; Mostafa et al., 2022). However, the volatile indole, an important intermediate during tryptophan biosynthesis, was decreased significantly in ‘Yujin2’ (Supplementary Table 3). Indole is a volatile compound emitted to a plant’s surroundings and functions as a remote signal, while indole-derived metabolites are mainly non-volatile and are embedded in many biological systems. Our results implied that the upregulation of these genes may facilitate the production of tryptophan and non-volatile derivatives, such as the biosynthesis of scent metabolites and the phytohormone auxin, and well as the coloring of yellow petals in ‘Yujin2’ (Cna’ani et al., 2018).

Noteworthy, methyl anthranilate, a VOC with grape-like and orange blossom odor that was substantially accumulated and considerably increased in ‘Yujin2’ compared to ‘Fengjin1’, is derived from the methylation of anthranilate in plants (Luo et al., 2019). Anthranilate, which is derived from chorismate *via* the AS enzyme complex, is an intermediate in tryptophan biosynthesis (Luo et al., 2019). The significantly upregulated Asα and Asβ in ‘Yujin2’ may be closely related to the increase of methyl anthranilate and the strong aroma of ‘Yujin2’ (Figure 9; Supplementary Table 12).

### Fatty acid derivative biosynthetic pathway was activated in ‘Yujin2’

Fatty acid derivatives also constitute a major class of flower VOCs, which are derived from the unsaturated C18 fatty acids linolenic and linoleic acid. The initiation of volatile fatty acid derivative biosynthesis is catalyzed by 9-and 13-lipoxygenase (LOX), which leads to the formation of 9-and 13-hydroperoxide intermediates, respectively. Then, 9-and 13-hydroperoxide lyases (HPLs), respectively, convert 9-and 13-hydroperoxides to volatile C9 and C6 aldehydes, which are reduced by alcohol dehydrogenases (ADHs) to C9 and C6 alcohols, and C6 alcohols are converted into esters by alcohol acyltransferase. In the current study, seven genes of LOXs, an HPL gene, and an ADH2 gene were significantly upregulated in ‘Yujin2’ (Figure 9; Supplementary Table 12). The upregulation of these genes involved in the fatty acid derivative biosynthetic pathway plays an important role in the accumulation of volatile C9 and C6 aldehydes and alcohols, as well as various esters, in ‘Yujin2’ (Muhlemann et al., 2014).

### Transcription factors play a crucial role in the regulation of the floral scent biosynthetic network

Orchestrated production of VOCs from these independent pathways requires coordinated transcriptional regulation of the floral scent biosynthetic network. To date, a number of TF families have been reported to regulate the production of VOCs in various plants, including MYB, bHLH, WRKY, ERF/AP2, bZIP, and NAC-type TFs (Qiao et al., 2021; Mostafa et al., 2022). For instance, ODORANT1 (DOD1), a member of the R_2_R_3_-type MYB family, regulates the synthesis of precursors in the shikimate pathway and the entry points to the phenylpropanoid and benzenoid biosynthetic pathways (Verdonk et al., 2005; Yoshida et al., 2018). In the current study, DOD1 and its predicted target gene EPSPS involved in shikimate pathway were both downregulated, which were consistent with the significantly decreased accumulation of benzenoids in ‘Yujin2’ (Figure 5; Supplementary Table 12). In *Osmanthus fragrans* flowers, WRKY TFs play important roles in regulating the biosynthesis of volatile monoterpenes (Ding et al., 2020). The WRKY-regulated NMD, a gene involved in monoterpenoid biosynthesis, was significantly upregulated, which may contribute to the monoterpenes production in ‘Yujin2’ (Figure 5; Supplementary Table 12). The plant-specific TF LFY is a regulator of early flower development and involved in activating the expression of floral organ identity genes (Wellmer and Riechmann, 2010). However, no information is available to show if LFY is involved in the floral scent regulation, which warrants further investigation. The identification of these differentially regulated TFs between ‘Yujin2’ and ‘Fengjin1’ will pave the way for investigating the regulatory functions of TFs in VOC biosynthesis in LJFs.

## Conclusion

**Figure 10:**
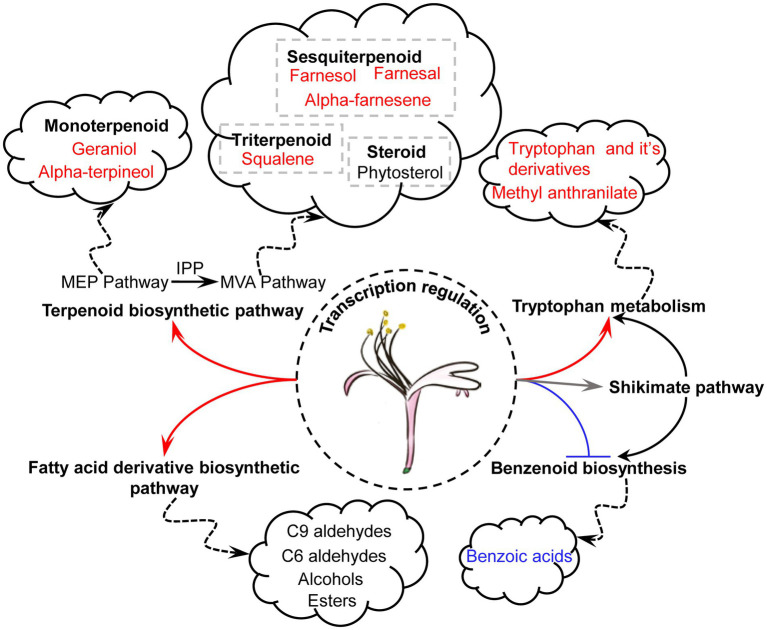
Schematic presentation of differentially regulated pathways and VOCs in LJFs of ‘Yujin2’ compared to ‘Fengjin1’. The biosynthetic pathways and accumulations of terpenoids, tryptophan and its derivatives, and fatty acid derivatives were upregulated, while the benzenoid biosynthesis was downregulated in ‘Yujin2’.

The regulatory mechanisms of the floral scents of *L. japonica* remain unclear. Integrative analyses of volatile metabolomics and transcriptomics of LJFs allowed identification of VOCs and pathways for the differences of floral aroma between ‘Yujin2’ and ‘Fengjin1’ (Figure 10). The differentially regulated pathways and VOCs in ‘Yujin2’ mainly include: (1) regulation of the TFs (MYB, WRKY, and LFY) for mediating the transcription of genes involved in VOC biosynthesis pathways; (2) activations of TES and GGPPS in MEP pathway for the increased odorous monoterpenoid, including alpha-terpineol and geraniol; (3) promoted HMGCS, HMGCR, MVK, FPS, and AFS1 in the MVA pathway for the induced odorous sesquiterpenoids, including farnesol, farnesal, and alpha-farnesene; (4) upregulated SQE1 and LUS in triterpenoid biosynthesis pathway and several genes involved in biosynthesis of phytosterols; (5) downregulation of biosynthesis and accumulation of benzenoids; (6) elevated tryptophan biosynthetic pathway for the increased tryptophan and methyl anthranilate; and (7) upregulation of the genes involved in the fatty acid derivative biosynthetic pathway for the accumulation of volatile C9 and C6 aldehydes and alcohols, and esters. Further functional analyses of the genes involved in VOC biosynthesis using molecular genetics and other approaches will promote advancements in understanding the regulatory mechanisms of floral scents in *L. japonica*.

## Data availability statement

The original contributions presented in the study are publicly available. This data can be found at: NCBI, PRJNA861870.

## Author contributions

JL and JY: conceptualization. XY, QS, and ZS: experiments and data analysis. XY and JY: writing—original draft preparation. CC, JL, XZ, and JL: writing—review and editing. All authors contributed to the article and approved the submitted version.

## Funding

This research was funded by the Science and Technology Major Project of Xinxiang City (ZD2020002 to JL), the China Postdoctoral Science Foundation and Doctoral Start-up Funding of Henan Normal University (2022M712143 and 5101049170191 to JY), and the National Natural Science Foundation of China (31970380 to JL).

## Conflict of interest

The authors declare that the research was conducted in the absence of any commercial or financial relationships that could be construed as a potential conflict of interest.

## Publisher’s note

All claims expressed in this article are solely those of the authors and do not necessarily represent those of their affiliated organizations, or those of the publisher, the editors and the reviewers. Any product that may be evaluated in this article, or claim that may be made by its manufacturer, is not guaranteed or endorsed by the publisher.

## Footnotes

^1^ https://pubchem.ncbi.nlm.nih.gov/

^2^ http://www.thegoodscentscompany.com/index.html

^3^ https://bigd.big.ac.cn/search/?dbId=gwh&q=honeysuckle

^4^ https://cloud.oebiotech.cn/task/detail/pca/

^5^ https://www.omicshare.com/tools/Home/Soft/volcano

^6^ http://www.kegg.jp/kegg/compound/

^7^ http://www.kegg.jp/kegg/pathway.html

^8^ http://planttfdb.cbi.pku.edu.cn/index.php

^9^ http://gtrd.biouml.org

^10^ https://string-db.org/

^11^ http://pfam.xfam.org

^12^ https://bigd.big.ac.cn/search/?dbId=gwh&q=honeysuckle

^13^ https://www.ncbi.nlm.nih.gov/cdd

^14^ https://www.ncbi.nlm.nih.gov/tools/primer-blast/
