# Supplementary material for: Integrated volatile metabolomic and transcriptomic analysis provides insights into the regulation of floral scents between two contrasting varieties of Lonicera japonica
Source: Front Plant Sci. 2022 Sep 12;13:989036. doi: 10.3389/fpls.2022.989036 (PMC9510994; doi:10.3389/fpls.2022.989036)
Supplement: Supplementary file 16 [file Presentation_1.PPTX]

## Slide 1
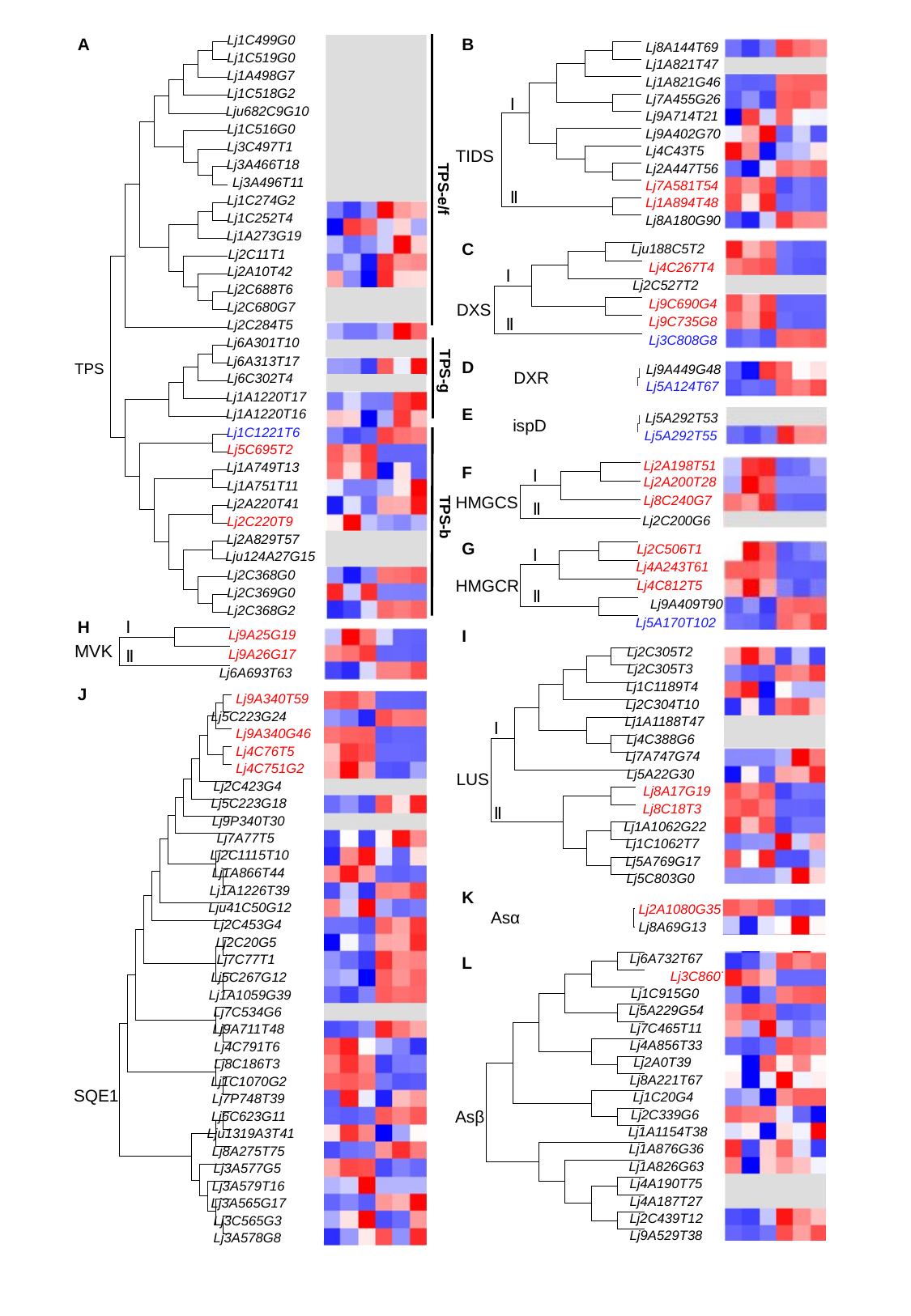

Lj1C499G0
 Lj1C519G0
 Lj1A498G7
 Lj1C518G2
 Lju682C9G10
 Lj1C516G0
 Lj3C497T1
 Lj3A466T18
 Lj3A496T11
 Lj1C274G2
 Lj1C252T4
 Lj1A273G19
 Lj2C11T1
 Lj2A10T42
 Lj2C688T6
 Lj2C680G7
 Lj2C284T5
 Lj6A301T10
 Lj6A313T17
 Lj6C302T4
 Lj1A1220T17
 Lj1A1220T16
 Lj1C1221T6
 Lj5C695T2
 Lj1A749T13
 Lj1A751T11
 Lj2A220T41
 Lj2C220T9
 Lj2A829T57
 Lju124A27G15
 Lj2C368G0
 Lj2C369G0
 Lj2C368G2
A
B
TPS-e/f
TPS-g
TPS-b
 Lj8A144T69
 Lj1A821T47
 Lj1A821G46
 Lj7A455G26
 Lj9A714T21
 Lj9A402G70
 Lj4C43T5
 Lj2A447T56
 Lj7A581T54
 Lj1A894T48
 Lj8A180G90
Ⅰ
TIDS
Ⅱ
C
 Lju188C5T2
 Lj4C267T4
 Lj2C527T2
 Lj9C690G4
 Lj9C735G8
 Lj3C808G8
Ⅰ
DXS
Ⅱ
D
TPS
Lj9A449G48
Lj5A124T67
DXR
E
Lj5A292T53
Lj5A292T55
ispD
 Lj2A198T51
 Lj2A200T28
 Lj8C240G7
 Lj2C200G6
F
Ⅰ
HMGCS
Ⅱ
G
 Lj2C506T1
 Lj4A243T61
 Lj4C812T5
 Lj9A409T90
 Lj5A170T102
Ⅰ
HMGCR
Ⅱ
Ⅰ
H
 Lj9A25G19
 Lj9A26G17
 Lj6A693T63
MVK
I
 Lj2C305T2
 Lj2C305T3
 Lj1C1189T4
 Lj2C304T10
 Lj1A1188T47
 Lj4C388G6
 Lj7A747G74
 Lj5A22G30
 Lj8A17G19
 Lj8C18T3
 Lj1A1062G22
 Lj1C1062T7
 Lj5A769G17
 Lj5C803G0
LUS
Ⅱ
J
 Lj9A340T59
 Lj5C223G24
 Lj9A340G46
 Lj4C76T5
 Lj4C751G2
 Lj2C423G4
 Lj5C223G18
 Lj9P340T30
 Lj7A77T5
 Lj2C1115T10
 Lj1A866T44
 Lj1A1226T39
 Lju41C50G12
 Lj2C453G4
 Lj2C20G5
 Lj7C77T1
 Lj5C267G12
 Lj1A1059G39
 Lj7C534G6
 Lj9A711T48
 Lj4C791T6
 Lj8C186T3
 Lj1C1070G2
 Lj7P748T39
 Lj6C623G11
 Lju1319A3T41
 Lj8A275T75
 Lj3A577G5
 Lj3A579T16
 Lj3A565G17
 Lj3C565G3
 Lj3A578G8
Ⅰ
Ⅱ
K
Lj2A1080G35
Lj8A69G13
Asα
 Lj6A732T67
 Lj3C860T0
 Lj1C915G0
 Lj5A229G54
 Lj7C465T11
 Lj4A856T33
 Lj2A0T39
 Lj8A221T67
 Lj1C20G4
 Lj2C339G6
 Lj1A1154T38
 Lj1A876G36
 Lj1A826G63
 Lj4A190T75
 Lj4A187T27
 Lj2C439T12
 Lj9A529T38
L
SQE1
Asβ

## Slide 2
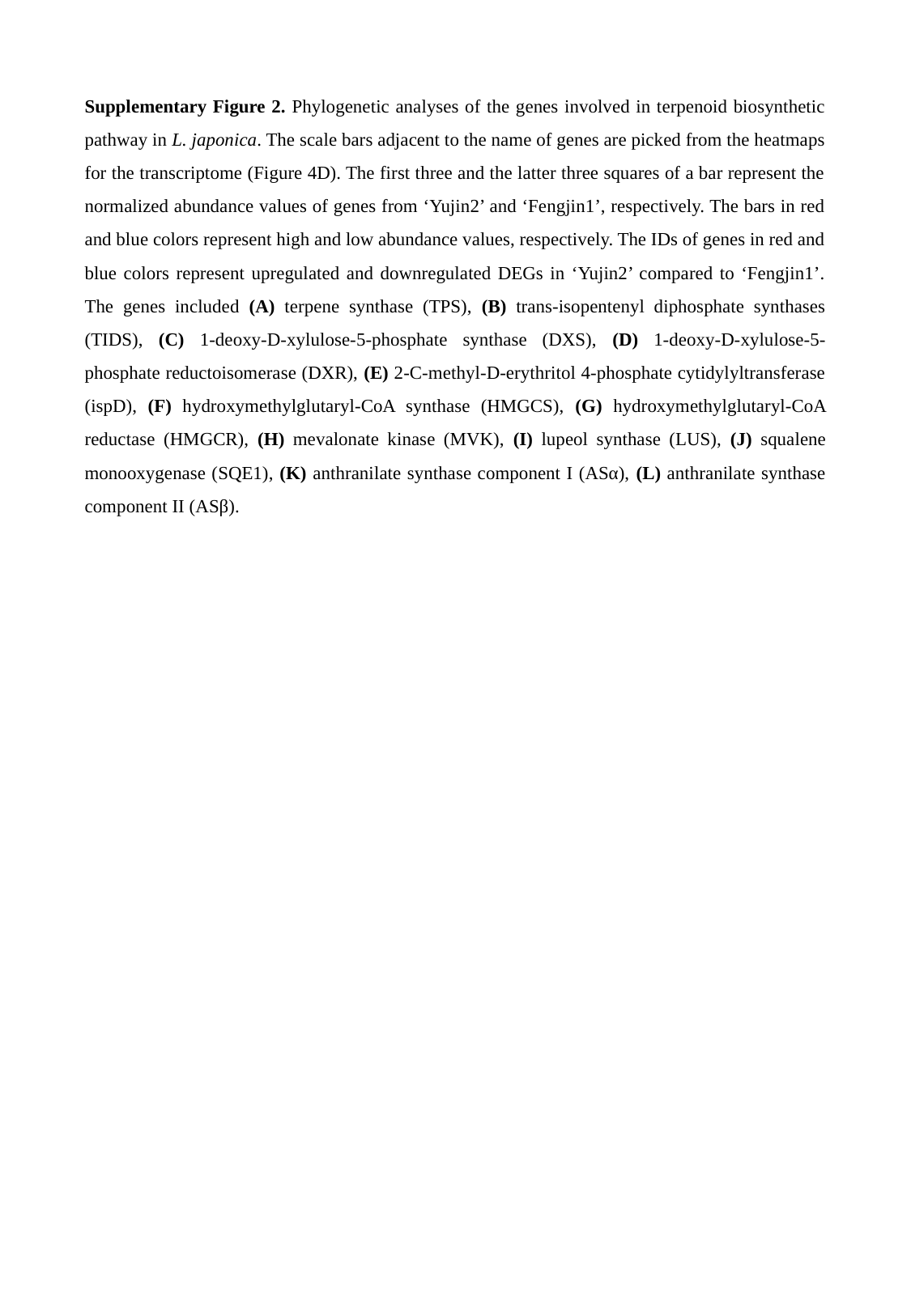

Supplementary Figure 2. Phylogenetic analyses of the genes involved in terpenoid biosynthetic pathway in L. japonica. The scale bars adjacent to the name of genes are picked from the heatmaps for the transcriptome (Figure 4D). The first three and the latter three squares of a bar represent the normalized abundance values of genes from ‘Yujin2’ and ‘Fengjin1’, respectively. The bars in red and blue colors represent high and low abundance values, respectively. The IDs of genes in red and blue colors represent upregulated and downregulated DEGs in ‘Yujin2’ compared to ‘Fengjin1’. The genes included (A) terpene synthase (TPS), (B) trans-isopentenyl diphosphate synthases (TIDS), (C) 1-deoxy-D-xylulose-5-phosphate synthase (DXS), (D) 1-deoxy-D-xylulose-5-phosphate reductoisomerase (DXR), (E) 2-C-methyl-D-erythritol 4-phosphate cytidylyltransferase (ispD), (F) hydroxymethylglutaryl-CoA synthase (HMGCS), (G) hydroxymethylglutaryl-CoA reductase (HMGCR), (H) mevalonate kinase (MVK), (I) lupeol synthase (LUS), (J) squalene monooxygenase (SQE1), (K) anthranilate synthase component I (ASα), (L) anthranilate synthase component II (ASβ).
