# Supplementary material for: Integrated volatile metabolomic and transcriptomic analysis provides insights into the regulation of floral scents between two contrasting varieties of Lonicera japonica
Source: Front Plant Sci. 2022 Sep 12;13:989036. doi: 10.3389/fpls.2022.989036 (PMC9510994; doi:10.3389/fpls.2022.989036)
Supplement: Supplementary file 17 [file Presentation_2.PPTX]

## Slide 1
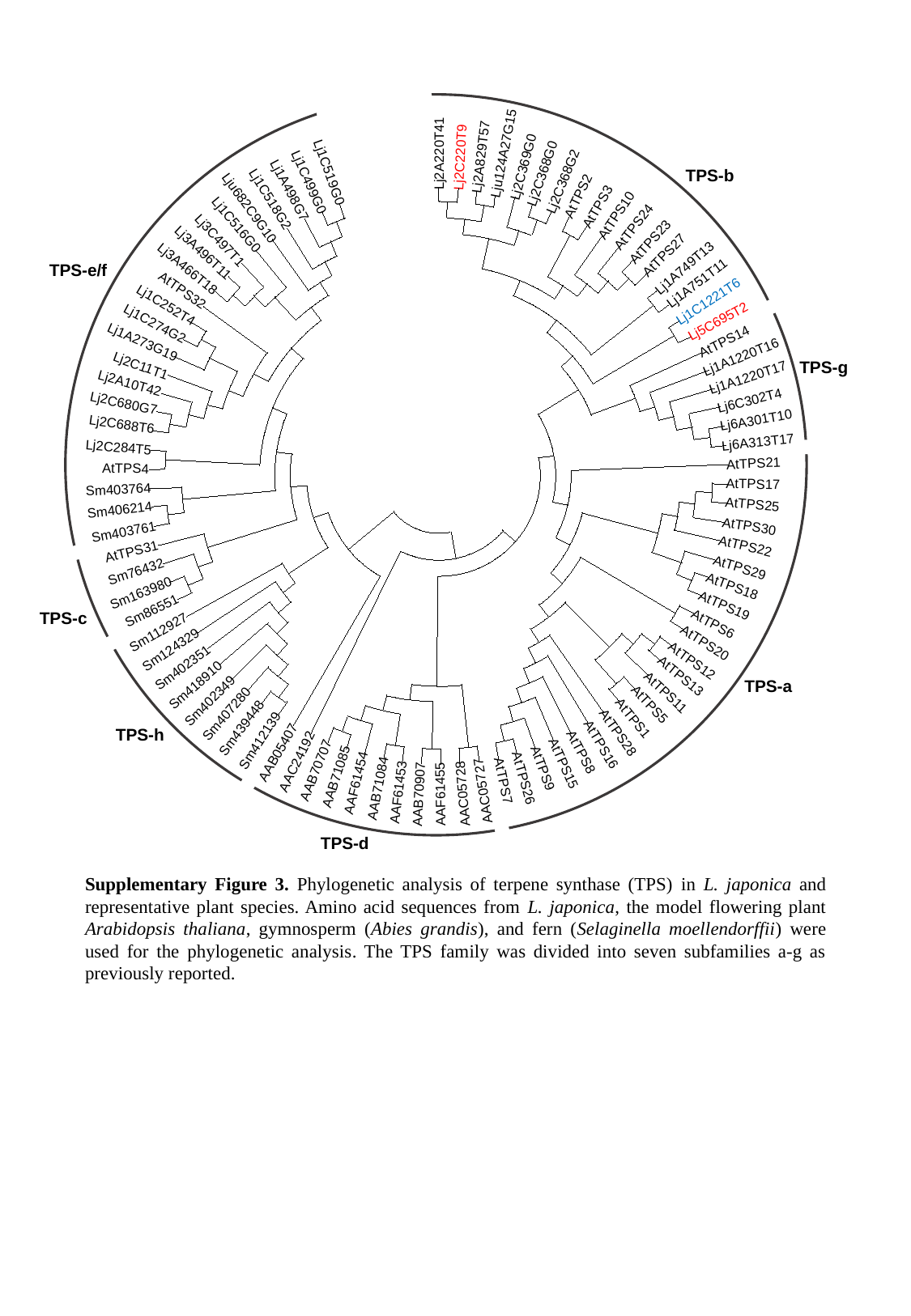

Lju124A27G15
Lj2A220T41
Lj2A829T57
Lj2C220T9
Lj2C369G0
Lj2C368G0
Lj2C368G2
AtTPS2
AtTPS3
AtTPS10
AtTPS24
AtTPS23
AtTPS27
Lj1A749T13
Lj1A751T11
Lj1C1221T6
Lj5C695T2
AtTPS14
Lj1A1220T16
Lj1A1220T17
Lj6C302T4
Lj6A301T10
Lj6A313T17
AtTPS21
AtTPS17
AtTPS25
AtTPS30
AtTPS22
AtTPS29
AtTPS18
AtTPS19
AtTPS6
AtTPS20
AtTPS12
AtTPS13
AtTPS11
AtTPS5
AtTPS1
AtTPS28
AtTPS16
AtTPS8
AtTPS15
AtTPS9
AtTPS26
AtTPS7
Lj1C519G0
Lj1C499G0
Lj1A498G7
Lj1C516G0
Lj3C497T1
Lj3A496T11
Lj3A466T18
AtTPS32
Lj1C252T4
Lj1C274G2
Lj1A273G19
Lj2C11T1
Lj2A10T42
Lj2C680G7
Lj2C688T6
Lj2C284T5
AtTPS4
Sm403764
Sm406214
Sm403761
AtTPS31
Sm76432
Sm163980
Sm86551
Sm112927
Sm124329
Sm402351
Sm418910
Sm402349
Sm407280
Sm439448
Sm412139
AAB05407
AAC24192
AAB70707
AAB71085
AAF61454
AAB71084
AAC05727
AAF61453
AAB70907
AAF61455
AAC05728
Lj1C518G2
Lju682C9G10
TPS-b
TPS-e/f
TPS-g
TPS-c
TPS-a
TPS-h
TPS-d
Supplementary Figure 3. Phylogenetic analysis of terpene synthase (TPS) in L. japonica and representative plant species. Amino acid sequences from L. japonica, the model flowering plant Arabidopsis thaliana, gymnosperm (Abies grandis), and fern (Selaginella moellendorffii) were used for the phylogenetic analysis. The TPS family was divided into seven subfamilies a-g as previously reported.
