# Supplementary material for: Integrated volatile metabolomic and transcriptomic analysis provides insights into the regulation of floral scents between two contrasting varieties of Lonicera japonica
Source: Front Plant Sci. 2022 Sep 12;13:989036. doi: 10.3389/fpls.2022.989036 (PMC9510994; doi:10.3389/fpls.2022.989036)
Supplement: Supplementary file 18 [file Presentation_3.PPTX]

## Slide 1
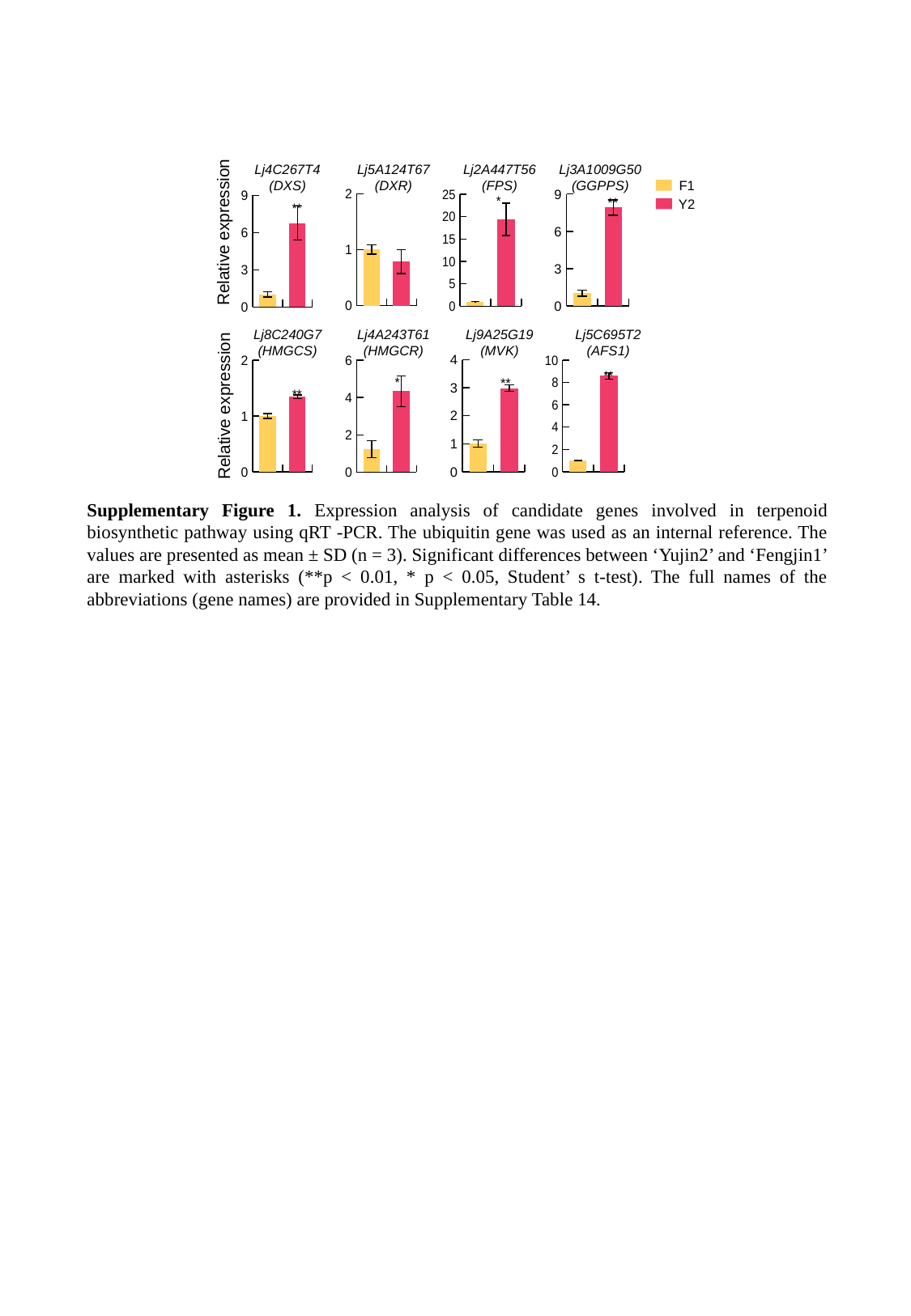

Relative expression
Lj4C267T4
(DXS)
Lj5A124T67
(DXR)
Lj2A447T56
(FPS)
Lj3A1009G50
(GGPPS)
### Chart
| Category | DXR |
|---|---|
| 9-F1 | 1.0023182525207426 |
| 9-Y2 | 0.7841974685662177 |
### Chart
| Category | FPS |
|---|---|
| 16-F1 | 1.000486498118212 |
| 16-Y2 | 19.388013161809045 |
### Chart
| Category | GGPS |
|---|---|
| 15-F1 | 1.0182351226629136 |
| 15-Y2 | 7.9065724194574605 |
### Chart
| Category | DXS |
|---|---|
| 7-F1 | 1.014259298628993 |
| 7-Y2 | 6.753101378376818 |F1
Y2
Lj8C240G7
(HMGCS)
Lj4A243T61
(HMGCR)
Lj9A25G19
(MVK)
Lj5C695T2
(AFS1)
Relative expression
### Chart
| Category | HMGCS |
|---|---|
| 21-F1 | 1.0006773062044865 |
| 21-Y2 | 1.3444051511938218 |
### Chart
| Category | HMGCR |
|---|---|
| 4-F1 | 1.234021970986699 |
| 4-Y2 | 4.3344942012011005 |
### Chart
| Category | MVK |
|---|---|
| 23-F1 | 1.0054364124441553 |
| 23-Y2 | 2.984258248391298 |
### Chart
| Category | AFS1 |
|---|---|
| 13-F1 | 1.0002787656162877 |
| 13-Y2 | 8.597391632684415 |Supplementary Figure 1. Expression analysis of candidate genes involved in terpenoid biosynthetic pathway using qRT -PCR. The ubiquitin gene was used as an internal reference. The values are presented as mean ± SD (n = 3). Significant differences between ‘Yujin2’ and ‘Fengjin1’ are marked with asterisks (**p < 0.01, * p < 0.05, Student’ s t-test). The full names of the abbreviations (gene names) are provided in Supplementary Table 14.
